# Inflammatory cytokines and mechanical injury induce post-traumatic osteoarthritis-like changes in a human cartilage-bone-synovium microphysiological system

**DOI:** 10.1186/s13075-022-02881-z

**Published:** 2022-08-18

**Authors:** Garima Dwivedi, Lisa Flaman, Begum Alaybeyoglu, André Struglics, Eliot H. Frank, Susan Chubinskya, Stephen B. Trippel, Vicki Rosen, Murat Cirit, Alan J. Grodzinsky

**Affiliations:** 1 Department of Biological Engineering, Massachusetts Institute of Technology, Cambridge, MA USA; 2 Department of Developmental Biology, Harvard School of Dental Medicine, Boston, MA USA; 3 Javelin Biotech, Woburn, MA USA; 4 Department of Clinical Sciences Lund, Orthopaedics, Faculty of Medicine, Lund University, Lund, Sweden; 5 Departments of Pediatrics, Orthopedic Surgery and Medicine (Section of Rheumatology), Rush University Medical Center, Chicago, IL USA; 6 Department of Orthopaedic Surgery, Indiana University School of Medicine, Indianapolis, IN USA; 7 Department of Electrical Engineering and Computer Science, Massachusetts Institute of Technology, Cambridge, MA USA; 8 Department of Mechanical Engineering, Massachusetts Institute of Technology, NE47-377, 500 Technology Square, Cambridge, MA 02139 USA

**Keywords:** Post-traumatic osteoarthritis, Inflammatory cytokines, Osteochondral-synovium coculture, Cartilage mechanical injury, Cartilage-bone-synovium metabolomics, Glycosaminoglycan, ARGS-aggrecan fragments, Microphysiological system

## Abstract

**Background:**

Traumatic knee injuries in humans trigger an immediate increase in synovial fluid levels of inflammatory cytokines that accompany impact damage to joint tissues. We developed a human in vitro cartilage-bone-synovium (CBS) coculture model to study the role of mechanical injury and inflammation in the initiation of post-traumatic osteoarthritis (PTOA)-like disease.

**Methods:**

Osteochondral plugs (cartilage-bone, CB) along with joint capsule synovium explants (S) were harvested from 25 cadaveric distal femurs from 16 human donors (Collin’s grade 0–2, 23–83years). Two-week monocultures (cartilage (C), bone (B), synovium (S)) and cocultures (CB, CBS) were established. A PTOA-like disease group was initiated via coculture of synovium explants with mechanically impacted osteochondral plugs (CBS+INJ, peak stress 5MPa) with non-impacted CB as controls. Disease-like progression was assessed through analyses of changes in cell viability, inflammatory cytokines released to media (10-plex ELISA), tissue matrix degradation, and metabolomics profile.

**Results:**

Immediate increases in concentrations of a panel of inflammatory cytokines occurred in CBS+INJ and CBS cocultures and cultures with S alone (IL-1, IL-6, IL-8, and TNF-α among others). CBS+INJ and CBS also showed increased chondrocyte death compared to uninjured CB. The release of sulfated glycosaminoglycans (sGAG) and associated ARGS-aggrecan neoepitope fragments to the medium was significantly increased in CBS and CBS+INJ groups. Distinct metabolomics profiles were observed for C, B, and S monocultures, and metabolites related to inflammatory response in CBS versus CB (e.g., kynurenine, 1-methylnicotinamide, and hypoxanthine) were identified.

**Conclusion:**

CBS and CBS+INJ models showed distinct cellular, inflammatory, and matrix-related alterations relevant to PTOA-like initiation/progression. The use of human knee tissues from donors that had no prior history of OA disease suggests the relevance of this model in highlighting the role of injury and inflammation in earliest stages of PTOA progression.

**Supplementary Information:**

The online version contains supplementary material available at 10.1186/s13075-022-02881-z.

## Introduction

Post-traumatic osteoarthritis (PTOA) is a major unsolved problem in modern musculoskeletal medicine. It afflicts over 75 million individuals worldwide [1–4]. It develops as a result of traumatic joint injury, usually involving anterior cruciate ligament (ACL) or meniscal tears, and the incidence of these injuries is increasing over time [2]. As PTOA progresses, fibrillation and loss of cartilage volume are accompanied by hyperplasia and fibrosis of the synovial membrane and joint capsule, subchondral bone sclerosis, and formation of bone osteophytes and cysts [3]. Unfortunately, surgical repair does not reduce the risk of PTOA progression [4], and persons who suffer from advanced PTOA are often too young to be suitable candidates for joint replacement. The etiopathogenesis of PTOA has not been elucidated and there is no known cure.

Up to 90% of ACL injuries are accompanied by osteochondral lesions as seen on MRI, suggesting that cartilage sustains high mechanical impact at the time of injury [5]. This acute overload can itself cause matrix alterations, cell death, and subchondral bone damage [6]. In addition, multiple studies report that inflammatory cytokines along with cartilage and bone markers are increased in patient synovial fluids as early as 24–48 h after knee injury [7–11]. Levels of IL-6, IL-8, TNF-α, IL-1β, IFNγ, and others can remain elevated up to 5 years after injury and are associated with proteolysis of aggrecan, type II collagen [7], and additional cartilage matrix molecules along with accompanying bone changes leading to fully developed PTOA [3].

In vivo animal models of OA/PTOA and in vitro studies of isolated cartilage and bone have revealed important insights into degradative processes relevant to PTOA progression [3]. Recently, more advanced in vitro coculture models have provided further approaches to achieve mechanistic understanding and platforms for drug discovery and drug delivery [12–14]. This is particularly useful for the earliest stages of PTOA, as clinical studies in humans typically focus on more advanced OA [3]. As PTOA is a disease of the total joint, including cartilage, bone, synovium, ligaments, and menisci [3], in vitro models focusing on single isolated tissues fail to incorporate crosstalk between tissues, which is critical to the pathophysiology of disease and evaluation of potential therapeutic targets [15]. Experiments utilizing cell monolayers or 3D cultures of cells in scaffolds are often unable to recapitulate the native biology of fully developed extracellular matrix-rich musculoskeletal tissues [16].

Previous in vitro studies have shown that mechanical injury to cartilage alone results in chondrocyte cell death [17, 18], increased matrix degradation [19], and altered chondrocyte biosynthesis [20]. However, given the combined effects of inflammation and mechanical trauma on the osteochondral unit in human PTOA pathogenesis, it would be most revealing to include joint capsule synovium tissue in an explant coculture system [14]. Due to the challenge of obtaining nearly normal human knee tissues, most previous human coculture studies have utilized tissues from knee arthroplasties. While such end-stage OA tissues may provide valuable insights, tissues from donors having no OA disease history might best enable study of cellular and mechanistic events at the earliest stages of PTOA development, to establish a foundation for discovery of biomarkers and therapeutic targets.

Therefore, the overall goal of the present study was to develop a human cartilage-bone-synovium (CBS) coculture system incorporating impact mechanical injury (INJ) to model early events in PTOA initiation and in an inflammatory environment. We hypothesized that this CBS+INJ coculture would mimic certain pathways and physiological mechanisms associated with traumatic joint disease mechano-biology in vivo. Our specific aims were (1) to establish and characterize the CBS model involving coculture of human knee osteochondral plugs with joint capsule synovium explants harvested from the same knees, (2) to incorporate a single injurious impact compression of the osteochondral plugs in a manner that mimics osteochondral lesion caused by acute knee injuries, and (3) to use this CBS+INJ system to study early PTOA-like initiation and progression, focusing on changes in cell viability, matrix composition and degradation, metabolomic readouts of individual cartilage, bone, and synovial tissues, and metabolomic changes associated with coculture of near-normal human osteochondral tissues with inflammatory synovium.

## Methods

### Tissue harvest and culture

**Fig. 1:**
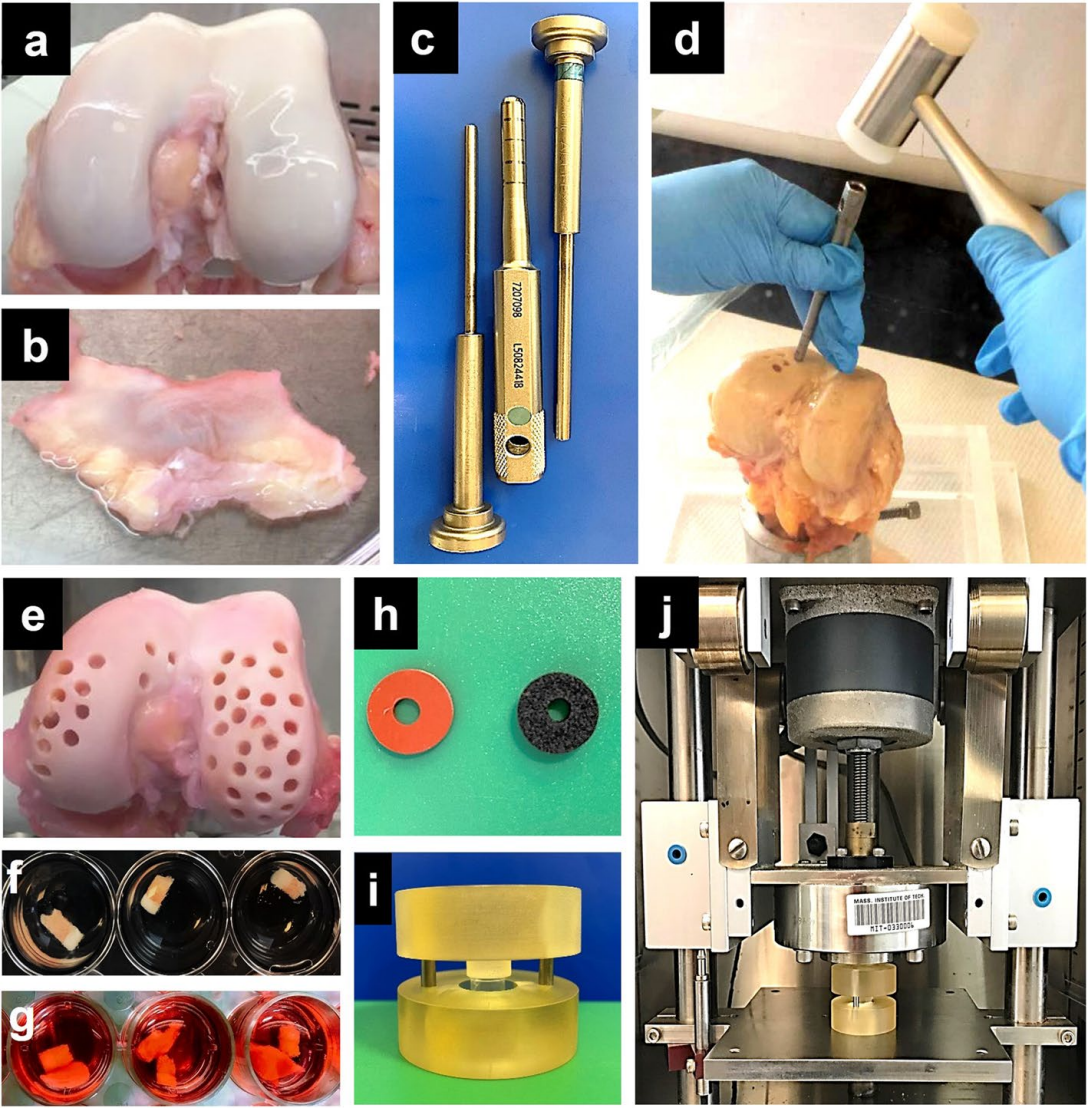
**a**–**g** Human cadaveric knees (**a**) and synovial joint capsule tissue (**b**) were obtained from 16 donors. Osteochondral plugs were harvested from the femoropatellar and condylar regions of human cadaveric distal femur using Mosaicplasty tool set (**c**). Synovial explants were cut from the synovial joint capsule obtained from the same donor. Cartilage-bone plugs (3.5 mm in diameter and up to 5 mm deep) were harvested (**d**, **e**) to set up cartilage-bone cocultures (**f**) and cartilage-bone-synovium cocultures (**g**). **i**, **j** Cartilage bone plug was held using rubber and foam rings (**h**) inside polysulfone injury chamber (**i**) to apply a single injurious unconfined compressive impact stress (15 MPa/s to peak stress of 5 MPa, held at 5MPa for 0.4 s, then unloaded at 15 MPa/s) on the cartilage surface in the incubator-housed loading apparatus (**j**) to simulate PTOA-like injury

Twenty-five knees from 16 human donors (age 23–83, 8 males, 8 females) were obtained postmortem from donor banks (Gift of Hope Organ and Tissue Donor Network, Itaska, IL; NDRI, Philadelphia, PA; LifeNet Health, Virginia Beach, VA) (see Supplementary Table S1 for list of joints and donor characteristics). All procedures were approved by MIT’s Committee on the Use of Humans as Experimental Subjects. Joint surfaces were graded using the modified Collins grading system [21] and all were grade 0–2 out of 4, where 0 indicates normal and 4 is end-stage OA. Since our objective was to initiate PTOA-like disease in joints that were initially as disease-free as possible, donors having any previous diagnosis or complaint of OA were excluded. Osteochondral plugs were harvested within 36–48 h of death from femoropatellar and condylar regions of the distal femur showing no apparent fibrillation or lesions on the cartilage surface (Fig. 1a). Full-thickness tissue inclusive of the synovium and fibrous joint capsule (together designated “Synovium-S” in Table S1) was obtained from the same donor knees (Fig. 1b). Surgical Mosaicplasty tool sets (Smith & Nephew, Cat No. 7207098, Fig. 1c) were used to harvest (Fig. 1d) cylindrical osteochondral plugs (3.5 mm diameter, 10–12 mm long (Fig. 1e, f)) that were distributed among experimental conditions such that femoropatellar and condylar regions were represented across all study groups (see Supplemental Methods A for harvesting details). The harvested osteochondral plugs (designated as cartilage-bone (CB) in Table S1) were first rinsed in PBS containing 1% streptomycin-amphotericin solution and then pre-equilibrated for 2–3 days in serum-free medium comprised of high-glucose DMEM [4.5 g/l] supplemented with 1% ITS (insulin-transferrin-selenium at 10μg/ml, 5.5μg/ml, and 5ng/ml, respectively; Sigma), 10 mM HEPES buffer, 0.1 mM nonessential amino acids, 0.4 mM proline, 20 μg/ml ascorbic acid, 100 U/ml penicillin G, 100 μg/ ml streptomycin, and 0.25 μg/ml amphotericin (pre-incubation medium) at 5% CO_2_, 37°C. Synovium tissue from the same donors was pre-incubated separately in pre-equilibration medium until the experiment started. After pre-equilibration, all subsequent 2–4-week mono- and cocultures used pre-incubation medium modified to contain low-glucose DMEM (1g/l) (incubation medium).

#### Monocultures

The subchondral bone (B) of each osteochondral plug was first trimmed to ~5mm using a Littauer Bone cutter (Fine Science Tools, Cat. No. 16152-12) to ensure consistent size of bone tissue explants. Full-thickness cartilage (*C*) was then separated from the underlying subchondral bone. Using a surgical scissors, the synovium (S, including native synovial and underlying fibrous capsular tissue) was cut into full-thickness explants (each 4–5 mm) to represent the effects of all cellular/molecular constituents on post-injury events. At the start of experiments, each separate C, B, and S explant was maintained in 500μl of incubation medium for 2–4 weeks (5% CO_2_, 37 °C).

#### Cocultures

Intact osteochondral plugs (*CB*) were each cultured in 1-ml incubation medium alone or cocultured with a single synovium explant (*CBS*) in 1.5-ml culture medium in separate studies (Fig. 1g). To address the variability CB plugs harvested from different regions across each joint surface, as well as variability in the cellular and matrix constituents of the S synovial explants, 5–6 CB and synovial explants taken across all harvested regions from each joint were assigned to each treatment condition. The medium was changed in all the cultures every 2–3 days for 2–4 weeks.

### PTOA disease model (CBS+INJ)

To simulate PTOA-like mechanical trauma, the cartilage surface of each osteochondral plug was subjected to a single injurious unconfined compression using an incubator-housed loading apparatus [22]. Each plug was first secured within three rings (two foam, one rubber, ~1.5mm thick with inside diameter 4mm) (Fig. 1h) to maintain the tissue upright. The plug-ring assembly was placed in a custom-built polysulfone injury/loading chamber, with cartilage surface facing upward (Fig. 1i), and the chamber then inserted into the loading apparatus (Fig. 1j). After injurious compression (15 MPa/s to peak stress of 5 MPa, held at 5MPa for 0.4 s, then unloaded at 15 MPa/s), the plugs were removed from the assembly, transferred back into culture medium, and a synovium explant was added to establish the coculture disease group (*CBS+INJ*). The medium was changed in every 2–3 days for 2 weeks (Supplementary Fig. S1).

### Tissue and spent media samples for assays

Tissue samples were collected weekly (Fig. S1) for histology and biochemical assays. Cartilage and bone were separated, weighed, and immediately frozen. Cartilage tissue digests were used to analyze sGAG and collagen content and biosynthesis rates (below). The medium from cultures collected at every change was used to assess sulfated GAG (sGAG) loss, inflammatory cytokine concentrations released by mono- and cocultures, and extracellular metabolomic profiles from mono- and cocultures.

### Viability of cartilage and bone

Viability of cartilage in mono- and cocultures was determined weekly using fluorescein diacetate/propidium iodide (FDA/PI, Sigma) as described [23]. For CB, cartilage was first separated from underlying bone; cross-sections 100–200μm thick were cut from the center of the cartilage (extending from top surface to deep zone) and treated with FDA (4mg/ml) and PI (1mg/ml) for 2 min, followed by PBS wash for 2 min. Images of slices were taken under blue and green filters at 4× magnification (Nikon fluorescence microscope) to determine viable (green) and dead (red) cells. The viability of bone tissue was determined using the MTT (3-(4,5-dimethylthiazol-2-yl)-2,5-diphenyltetrazolium bromide; Sigma M5655) method [24]. Bone explants were incubated in 10 ml of a 5-mg/ml MTT sterile solution in the dark at 37°C with 5% CO_2_ for 4 h. The intensity of purple color resulting from formation of formazan by metabolically active cells was visually inspected to assess viability.

### Inflammatory profile

Tissue release of cytokines to the medium was assessed on days 2, 4, 7, 9, 11, and 14. Ten cytokines relevant to human joint injury [7], including 7 pro-inflammatory (IL-1β, 1L-2, IL-6, IL-8, IL-12p70, IFN-γ, TNF-α) and 3 anti-inflammatory (IL-4, IL-10, IL-13), were analyzed using the Human V-Plex Plus proinflammatory 10 spot panel kit from Meso Scale Discovery (cat no. K15049D; Rockville, MD) capable of measuring cytokines from cell culture supernatants, following manufacturer’s directions. Spent media samples from 5–6 technical replicates were pooled for each time point to create one sample per time point for each donor. Pooled samples were undiluted, except 10× dilutions were needed for IL-6 and IL-8.

### Histology

Osteochondral tissues were fixed in 10% neutral buffered formalin for 2 days, decalcified, paraffin-embedded, and sectioned longitudinally; fixed synovial tissues were cross-sectioned. Deparaffinized and rehydrated osteochondral sections from conditions CB, CBS, and CBS+INJ were stained with hematoxylin and eosin (H&E — to assess structure and cellularity) and Toluidine-Blue (to visualize spatial distributions of sGAG within cartilage).

### Biochemical analyses

#### sGAG and total protein

Synthesis of sGAG and total protein was assessed by supplementing the culture medium with 20 μCi/ml [^35^S]-sulfate and 10 μCi/ml [^3^H]-proline (Perkin Elmer, Norwalk, CT), respectively, for 24 h (*n* = 3/group). Following 4 washes to remove free label, cartilage was separated from bone (in coculture groups) and wet weight (WW) taken for each cartilage and bone sample. Bone tissue was first flash-frozen in liquid nitrogen and crushed immediately into a fine powder. Bone tissue powders and cartilage were digested in 500 μl proteinase K solution (2mg/ml in 50mM Tris-HCl, 1mM CaCl_2_, pH8) (Roche, Indianapolis, MN) at 56°C for 16 h. [^35^S]-sulfate and [^3^H]-proline incorporation in cartilage digests were measured via liquid scintillation counting (Perkin Elmer) and normalized to wet weights. sGAG content of cartilage tissue digests and spent media was determined using the dimethylmethylene blue (DMMB) dye-binding assay [25]. Tissue digests were hydrolyzed and dried to assess the total collagen content using the hydroxyproline (OHP) assay [26] and normalized to wet weights.

#### ARGS-aggrecan and CTX-II

Spent media samples (*n* = 5–6 technical replicates) from each biological replicate (*N* = 3–6 donors) for each treatment condition were pooled for day 2, 7, and 14 time points to create one sample per donor per time point for both ARGS-aggrecan neoepitope and C-telopeptide fragments of type *II* collagen (CTX-II) fragments. ARGS-aggrecan, a biomarker of cartilage aggrecanase degradation, was assessed using an in-house electrochemiluminescence assay on the Meso Scale discovery platform [27]; see Supplementary Methods B for additional details. C-telopeptide fragments of type II collagen (CTX-II), a biomarker of cartilage degradation, were assessed using a Sandwich-ELISA following assay instructions (IDS, cat no. AC-08F1, Gaithersburg, MD) [28]. All medium samples were run in duplicates at the same dilution.

### Measurement of untargeted metabolomics

Metabolomics studies focused on circulating metabolites and were performed using spent media samples from monocultures and CBS cocultures of donors #1–3, 7, 11–14, and 16. Spent media samples from three biological replicates were used for donors #11 and 12, while for donors #1–3, 7, 13–14, and 16, spent media were pooled from multiple wells, on culture days 2 and 7, to focus on early alterations in circulating metabolites. Samples were shipped for analysis to Human Metabolome Technologies America, Inc. (HMT; Boston, MA, USA). Sample preparation was performed using the previously described protocol [29]. Metabolome analysis was performed using capillary electrophoresis time-of-flight mass spectrometry (CE-TOFMS) in two modes for cationic and anionic metabolites. A total of 116 metabolites were detected based on HMT’s standard library.

### Statistical analyses

Data for sGAG release, collagen content, and biosynthesis of sGAG and total protein were analyzed using the linear mixed-effects model with human donor as a random factor, followed by Tukey’s post hoc test for pairwise comparisons, performed using Prism GraphPad. For cytokine release, group differences in the total amount released (over 14 days) were analyzed using a donor-matched mixed-effects model (REML). False discovery rate (FDR, Benjamini-Hochberg method) was used to adjust for multiple comparisons. For ARGS-aggrecan and CTX-II release, data were grouped by treatment condition (C, CB, CBS, CBS+INJ) or days [2, 7, 14] and analyzed using Mann-Whitney *U* (Wilcoxon rank sum) tests via MATLAB. Metabolomics data were analyzed using principal component and hierarchical clustering analyses along with ANOVA to determine the significant differences in the levels of metabolites, using MetaboAnalyst via R. *p*-values, or in some cases FDR-adjusted *p*-values < 0.05, were considered statistically significant (see Supplementary Tables 1, 2, 3, 4 and 5 for additional details).

## Results

### Culture set up and viability

**Fig. 2:**
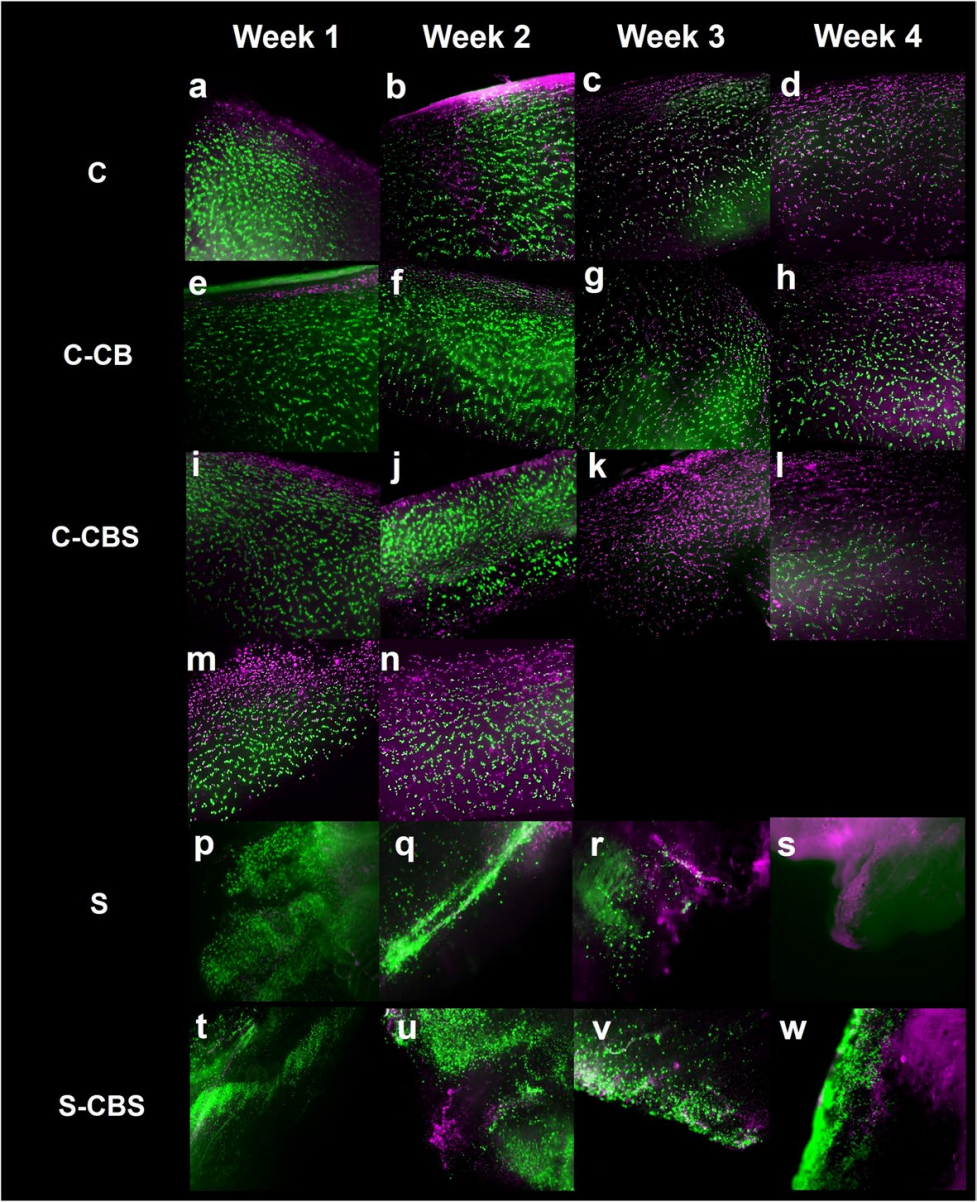
**a**–**l** Representative live-dead images from donor #1 tissues using FDA/PI revealed that chondrocyte viability was maintained in the cartilage tissue up to 3 weeks in C monoculture (**a**–**c**) and CB coculture (**e**–**g**). Severe loss in viability was observed in CBS cocultures (**i**–**l**) by the third week of culture (**k**, **l**). **m**, **n** Donor # 14 showed that the impact of mechanical injury was exacerbated by inflammatory environment, causing extensive cell death starting week 1 of culture in the CBS+INJ group; hence, viability was not assessed at week 3 and week 4 in this CBS+INJ group. **p**–**w** Donor #1 synovium tissue showed maintenance of synovium viability by 3 weeks for synovium monoculture (**p**–**r**) and in CBS coculture (**t**–**v**). Increased cell death in synovium was observed by week 4 in mono- (**s**) and CBS coculture (**w**). C, cartilage monoculture; S, synovium monoculture; C-CB, cartilage from osteochondral coculture; C-CBS, cartilage from osteochondral plugs cocultured with synovium; C-CBS + INJ, cartilage from mechanically injured osteochondral plugs cocultured with synovium; S-CBS, synovium explant from osteochondral plugs cocultured with synovium

Viable mono- and cocultures were established from human cartilage, bone, and synovial tissues. Representative live-dead images across donor tissues revealed minimal chondrocyte death in cartilage alone and in osteochondral plugs during the first 3 weeks of culture (Fig. 2a–h), and chondrocyte death was typically limited to the cartilage superficial zone. Increased chondrocyte death was observed in all groups by week 4 including C and CB, which is to be expected even in healthy tissues because of inherent limitations of in vitro culture (including limited nutrient availability and waste exchange, and absence of normal mechanical forces) (Fig. 2d, h, l). In CBS cocultures, most chondrocytes were dead by the end of week 3 (Fig. 2k). However, compared to C-monocultures in the absence of mechanical injury and inflammation, the most severe loss in chondrocyte viability was observed in CBS+INJ cultures as early as week 1. Since the combination of mechanical injury and synovium coculture adversely affected chondrocyte viability (Fig. 2m, n), the (CBS+INJ) treatment group was studied for only 2 weeks. Viability was also assessed in the synovium tissue, both in mono- and CBS cocultures; live cells were predominant by the end of week 3, though some dead cells were scattered throughout (Fig. 2p–r, t–v). Increased cell death was observed at week 4 (Fig. 2s, w). Bone tissue incubated in MTT solution for 4 h at the end of experiments showed purple staining indicative of live cells (data not shown).

### Cytokine release was predominant in cultures that included synovium

**Fig. 3:**
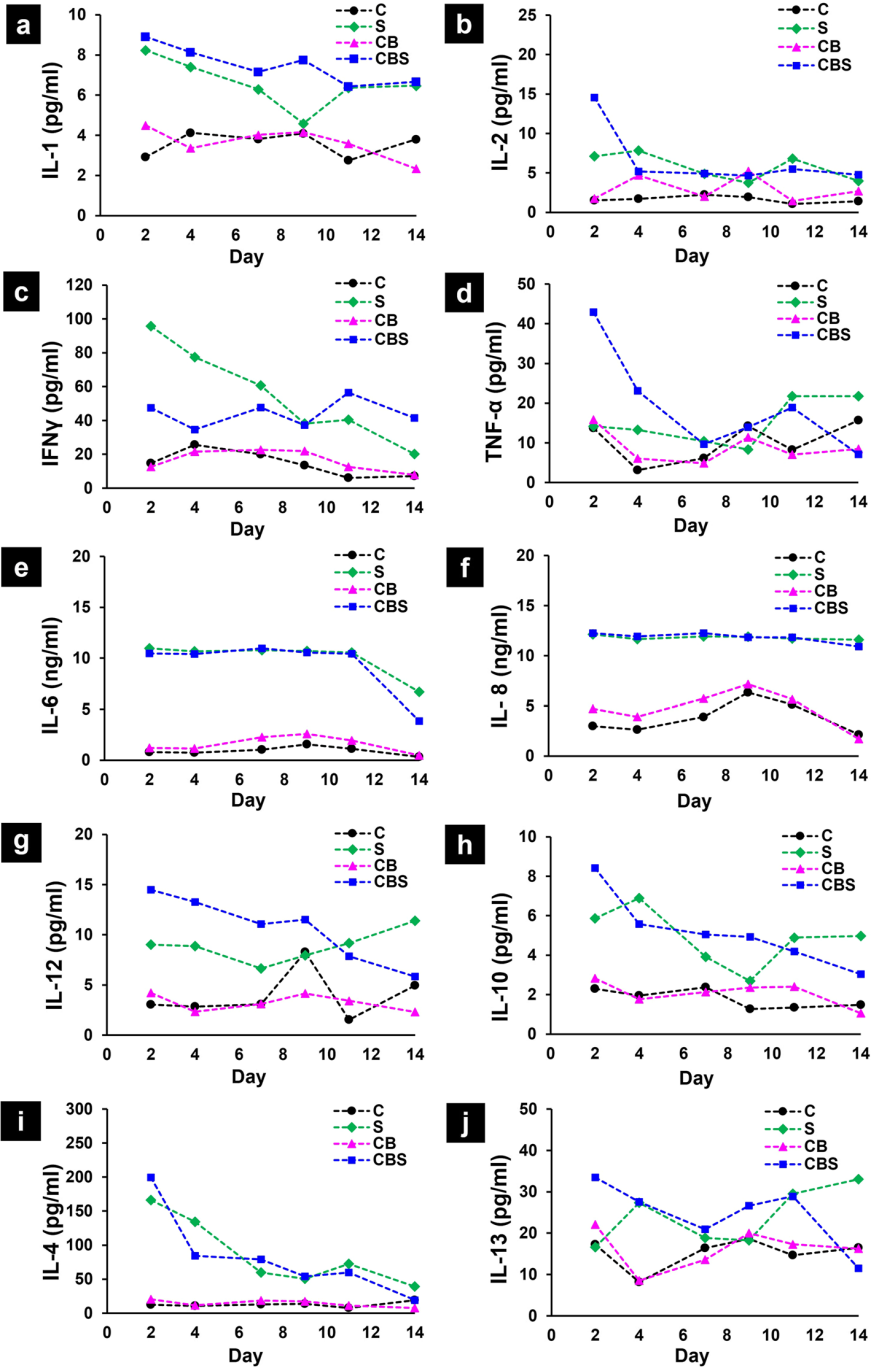
**a**–**g** Concentrations of pro- (**a**–**g**) and anti- (**h**–**j**) inflammatory cytokines released from donor #7 sample groups to the medium during 14-day cultures. Concentrations of pro-inflammatory cytokines were higher in the synovial monocultures and CBS cocultures, suggesting that the synovium tissue was the primary source of these cytokines. C, cartilage monocultures; CB, intact osteochondral plugs; CBS, osteochondral plugs cocultured with synovium; S, synovium explant monocultures

The release of 10 cytokines into the medium was measured over the culture duration from separate treatment groups using tissues from all 16 donors (see Table S1 for the listing of treatment groups tested for each donor based on available tissues harvested from each knee). A representative example (donor #7, Fig. 3) strongly suggests that cytokines (IL-1β, IL-2, IL-12, IFNγ, TNF-α, IL-6, IL-8) are released to the medium predominantly by conditions that include synovium (i.e., *S* monoculture and *CBS* coculture). The levels observed in CBS cocultures were generally similar to those in synovium monocultures while far lower levels were observed in cultures of cartilage alone (*C*) or osteochondral plugs (*CB*). A common occurrence was an initial elevated release of cytokines in the first week, followed by a decline to lower levels for the rest of the culture (Fig. 3).

**Fig. 4:**
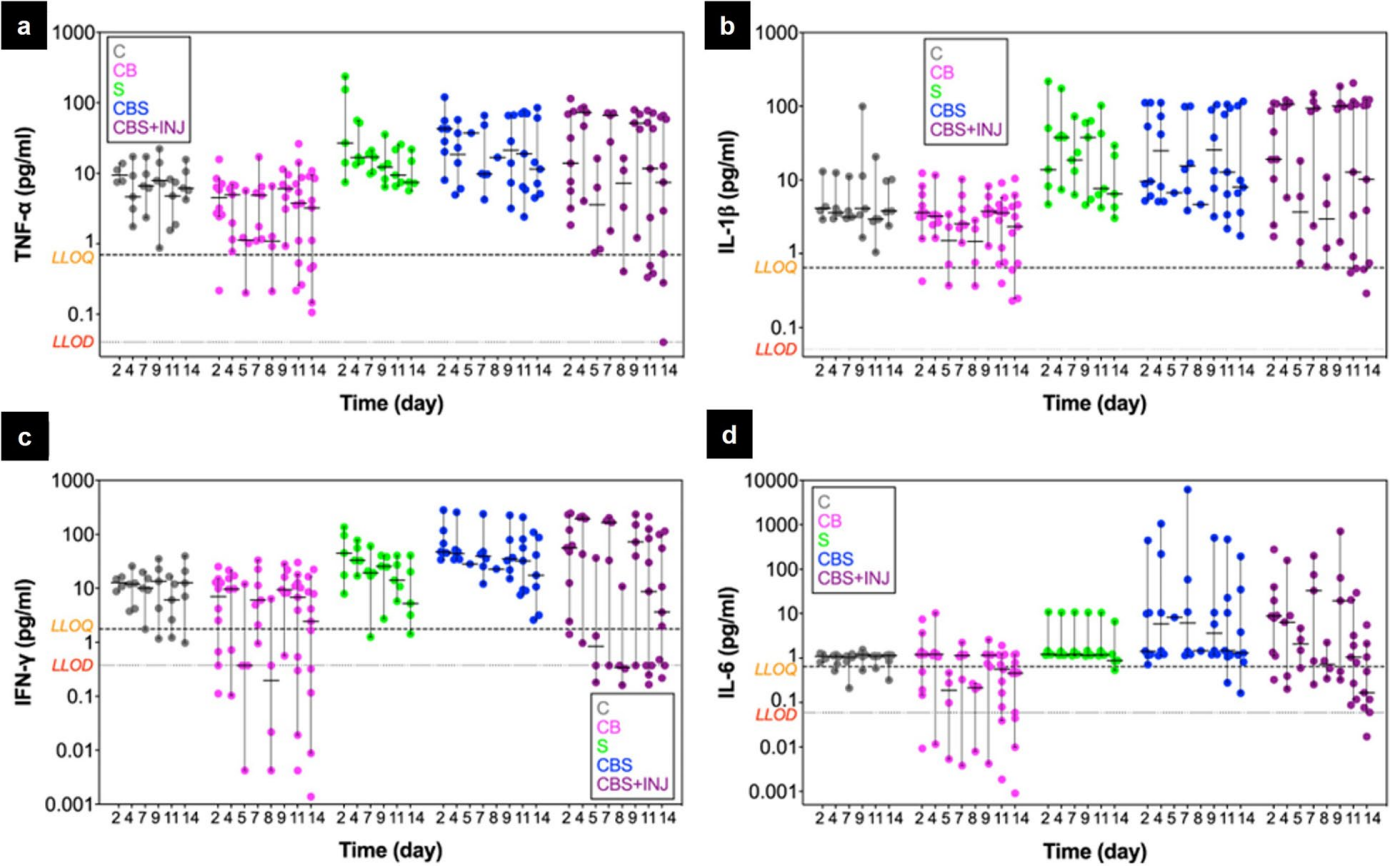
Concentrations of TNF-α (**a**), IL-1β (**b**), IFN-γ (**c**), and IL-6 (**d**) released into media during the 14-day culture period from all donors. Each data point represents one pooled sample from the 5–6 technical replicates at each time point for each donor, with the median and 95% CI displayed by black lines over the data points (see Table S1, “Inflammation” for the list of donors utilized for each treatment group). For each cytokine, the manufacturer’s LLOQ and median LLOD (Table S2) are shown in dashed and dotted horizontal lines, respectively. Measured concentration values of 0 pg/ml were replaced by median LLOD for visualization purposes. C, cartilage monocultures; CB, intact osteochondral plugs; CBS, osteochondral plugs cocultured with synovium; S, synovium explant monocultures; CBS + INJ, mechanically injured osteochondral plugs cocultured with synovium

However, these cytokine concentrations varied widely from donor-to-donor, as shown in Fig. 4 for release of TNF-α, IL-1β, IL-6, and IFN-γ to the media at all time points during 2-week cultures from all donors. Cytokine levels were typically highest at early times in culture, though they remained elevated in several donors. Levels were significantly higher (e.g., TNF-α, CB vs CBS and CB vs CBS+INJ, *p* = 0.0016 and 0.0105, respectively) in groups with synovium (S, CBS, CBS+INJ; statistical analyses summarized in Tables S2 and S3), while levels for cartilage monoculture (C) and osteochondral plugs (CB) were lower by 10–100-fold for most donors (Fig. 4).

### Histological observations

**Fig. 5:**
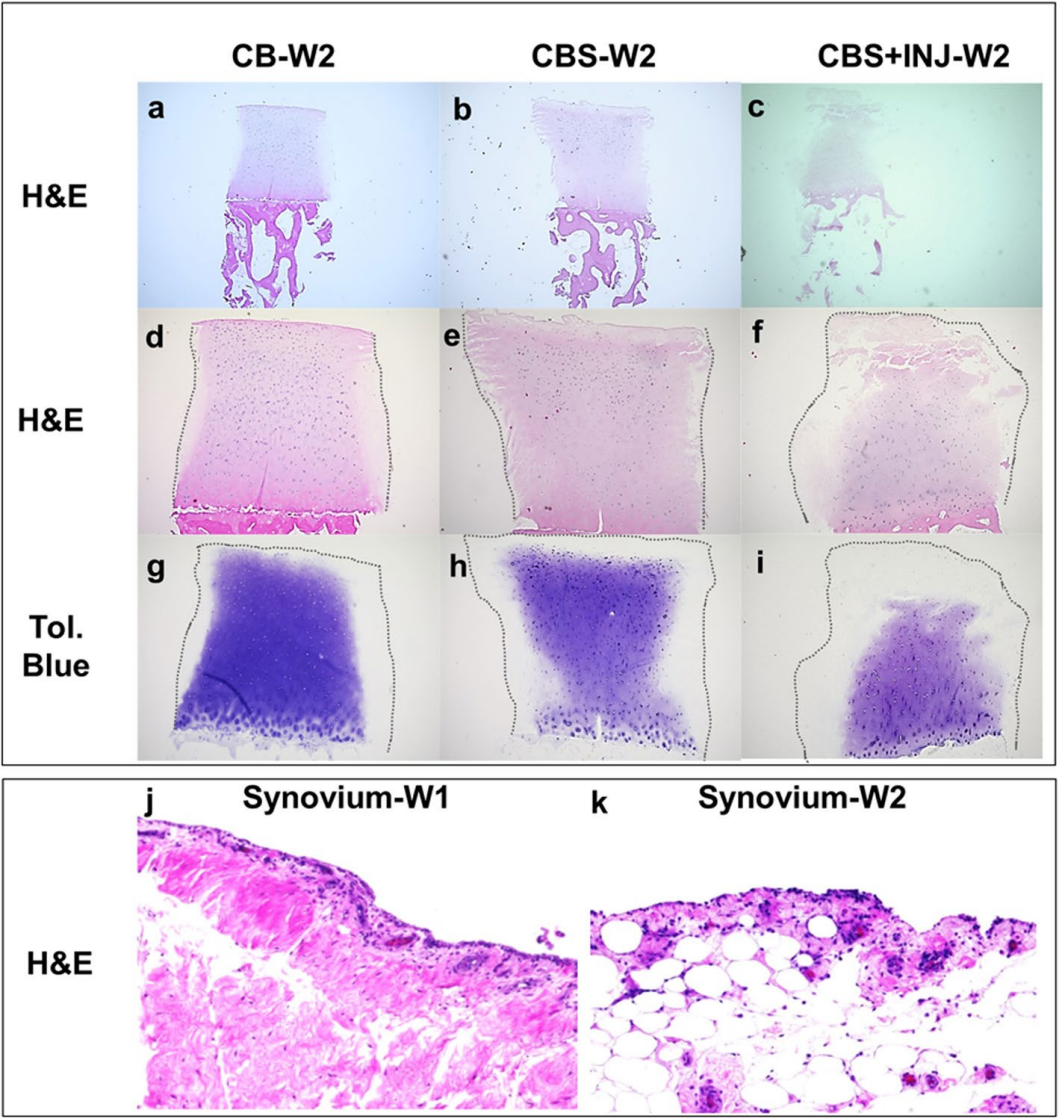
**a**–**f** H&E- and **g**–**i** Toluidine blue-stained sections from osteochondral plugs. Representative sections from donors #7 and 14 show **a** an osteochondral plug (*CB*) after 2 weeks (W2) in culture compared to **b** CB cocultured with synovium (S), i.e., *CBS*; **c** CB after mechanical injury cocultured with S (*CBS+INJ*). Representative sections from donor #7 show initial matrix depletion in cartilage of CB plugs (**d**, **g**). Coculture of osteochondral plugs with synovium (*CBS*) led to increased disruption of cartilage matrix (**e**) and further loss of GAG from the exposed sides and surface of cartilage (**h**). Further matrix disruption, loss of cellularity (**f**), and GAG (**i**) were observed in cartilage exposed to injury and coculture with synovium (donor #14) (*CBS + INJ*). **j**, **k** H&E-stained sections of synovium (donor #13) showing cells in synovial lining (2–3 layers) in both fibrous (**j**) and adipose (**k**) forms. Please note that **d**–**f** are enlargements of **a**–**c**. W, week

Histological sections focused on the appearance of osteochondral plugs cultured alone for 2 weeks compared to effects of coculture and mechanical injury. In the CB group, the surface of the cartilage tissue appeared smooth, and the cartilage structure was mostly intact (Fig. 5d). Some loss of cellularity was seen, which may have occurred due to the exposed surface of the radial periphery (Fig. 5a, d). In contrast, the cartilage surface appeared visibly damaged in conditions CBS (Fig. 5b, e) and CBS+INJ (Fig. 5c, f), especially the latter. CBS+INJ tissues also showed signs of hypocellularity, indicated by large areas without cells. The cartilage in the CBS+INJ group also showed swelling in the middle zone, suggesting loss of matrix structural integrity (Fig. 5f). Vertical fissures were observed in only a few samples from the CBS+INJ group, caused by mechanical injury (not shown). Increased loss of sGAG from cartilage (revealed by toluidine blue staining) was observed in CBS cultures (Fig. 5h) compared to cartilage in CB with no synovium (Fig. 5g). sGAG loss was further exacerbated in CBS+INJ and even more pronounced in the radial periphery extending into the deep zone of cartilage (Fig. 5i). H&E sections were obtained from fibrous and adipose forms of synovial tissues showing the intimal layer as well as the deeper layers of the tissue (e.g., Fig. 5j, k), but with no marked changes between groups.

### Increased release to medium of sGAG and ARGS, but not CTX-II over 2 weeks

**Fig. 6:**
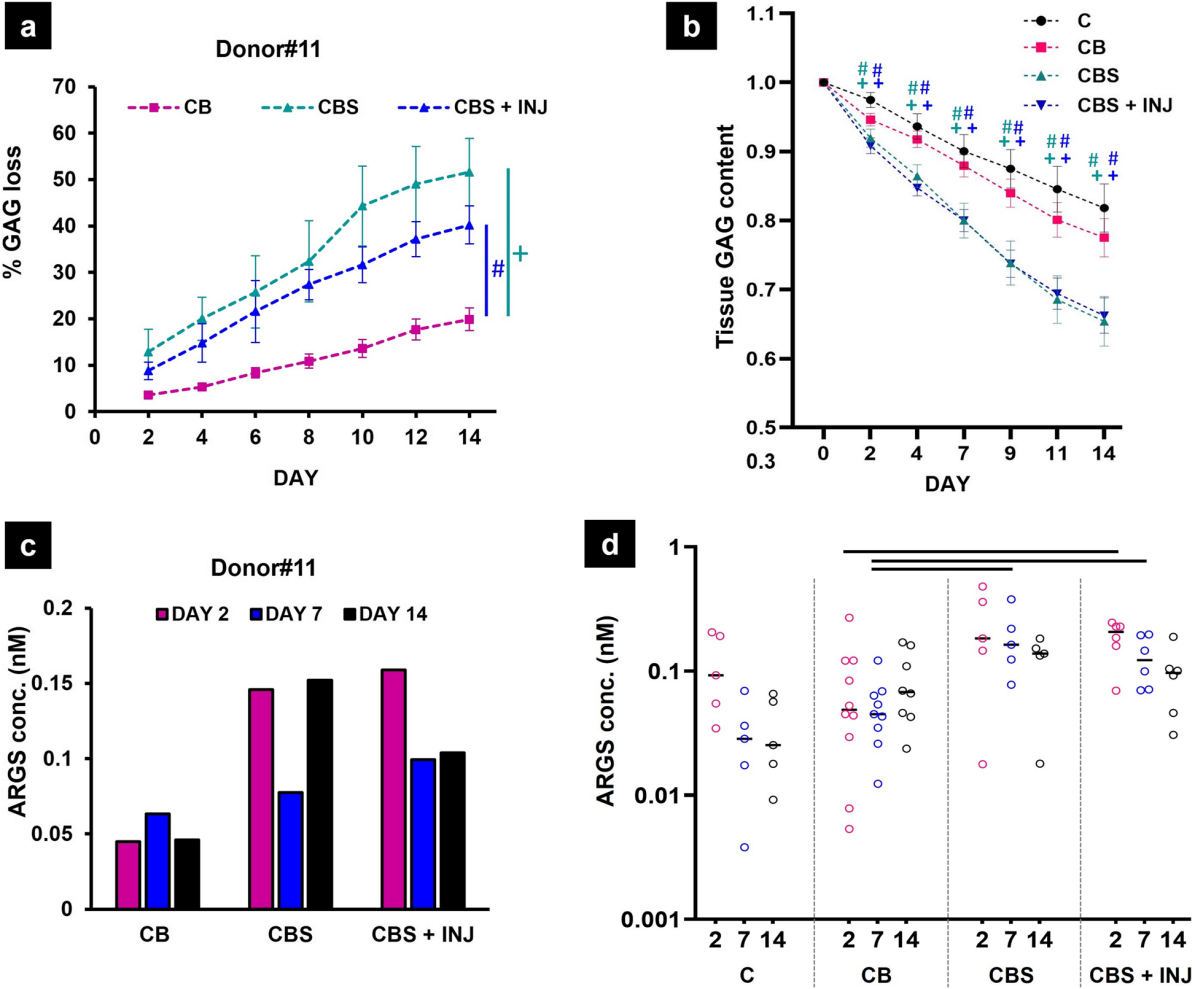
**a** Cumulative sGAG loss as a percent of total initial tissue GAG versus time, from representative donor #11, was significantly higher on day 14 in CBS (+) and CBS+INJ (#) groups versus CB. Data are mean ± SD of 5–6 technical replicates at each time point. **b** Tissue GAG (sGAG remaining in cartilage) for all donors for each treatment condition showed significantly higher loss in the presence of synovium explants (i.e., CBS or CBS+INJ) versus their absence (C or CB). Average values were first obtained for the 5–6 technical replicates at each time point for each donor; data are the mean cross all donors ± 95% C.I. Significant differences were observed in GAG content of C vs CBS (green+) and C vs CBS+INJ (blue+) from day 2. Tissue GAG was significantly lower starting day 2 in CBS vs CB (green#) and CBS+INJ vs CB group (blue#). Refer to Supplementary Table S4 for pairwise p values. **c** Release of ARGS-aggrecan fragments from representative donor #11 tissues on days 2, 7, and 14 to assess aggrecanase-mediated aggrecan degradation. Each bar represents the mean of pooled spent media samples obtained from *n* = 5–6 technical replicates. **d** Concentrations of ARGS-aggrecan fragments released to the medium from all donors on days 2, 7, and 14. Compared to CB, significantly higher levels of ARGS-aggrecan fragments were released in CBS and CBS+INJ groups in the early phase of culture (i.e., days 2 and 7; marked by horizontal lines). Each circle represents the mean of pooled spent media samples obtained from 5–6 technical replicates for a given donor; the bar is the median value for all donors assessed for each treatment condition at days 2, 7, and 14 (see Table S1-Biochemistry). C, cartilage monocultures, *N* = 5; CB, CB cocultures, *N* = 10; CBS, CBS cocultures, *N* = 5; CBS + INJ, disease group, *N* = 6

Total release of GAG from tissues, assessed qualitatively in the spatial aspects of histological observations (e.g., Fig. 5g–i), was also measured quantitatively as sGAG released to the medium (using the DMMB assay) from all donor tissues at all time points and culture conditions as listed in Table S1. Cumulative GAG loss (as a percent of total initial tissue GAG) was typically greater over 14 days in CBS and CBS+INJ culture compared to C and CB, exemplified in data from donor #11 (*p* < 0.05; Fig. 6a) (*n* = 5–6 technical replicates at each time point; monoculture C was not tested in donor #11). To account for donor variability when analyzing GAG loss from all donors, data were normalized as tissue GAG content (i.e., the amount of GAG remaining in cartilage at any time point normalized to total GAG (cartilage tissue + medium) at that time (Fig. 6b). Loss of tissue GAG was accelerated in CBS and CBS+INJ groups, compared to either C or CB, showing significant release as early as day 2 for C (*p* < 0.0001) and day 4 for CB (*p* < 0.0001) (Fig. 6b and Supplementary Table S4). The concentration of ARGS-aggrecan fragments released to the medium was determined at days 2, 7, and 14 in pooled spent media samples, shown for donor #11 (Fig. 6c) and then all donors (Fig. 6).

While there was considerable donor-to-donor variability, the release of ARGS-aggrecan fragments was significantly greater in CBS and CBS+INJ groups compared to CB cultures (*p* < 0.0011; *p* < 0.0001, respectively), as early as day 2 (statistical analyses summarized in Supplementary Table S5).

Biosynthesis rates for sGAG and total protein (via radiolabel incorporation) showed no significant differences between groups (Supplementary Fig. S2a, b).

No differences between groups were found for total tissue collagen content (hydroxyproline) or levels of released CTX-II fragments during the 2-week culture (Supplementary Fig. S2c, d).

### Monocultures have distinct circulating metabolic signatures

**Fig. 7:**
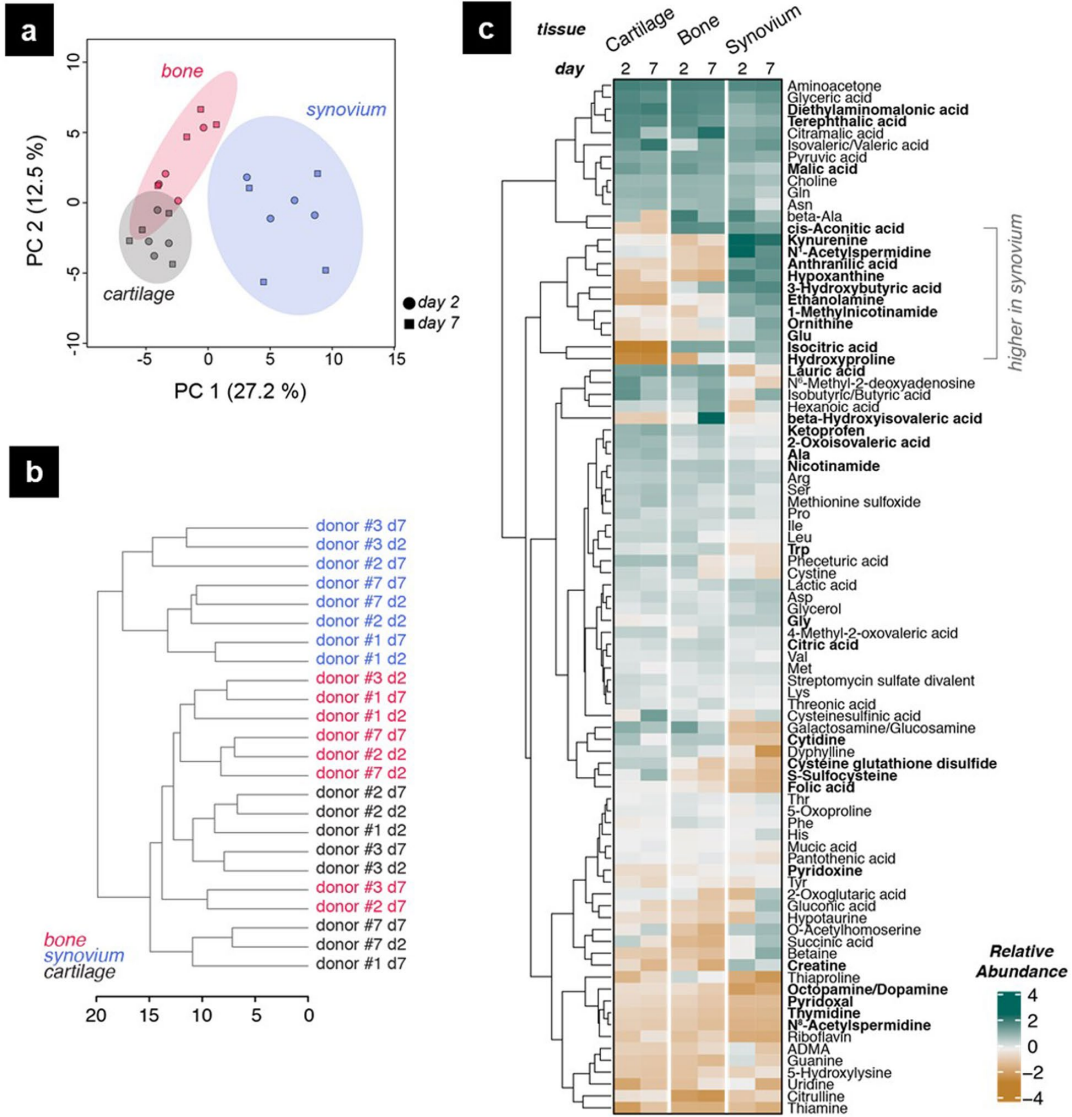
Circulating metabolites measured in cartilage, bone, and synovium monocultures from donors #1, 2, 3, and 7. **a** Principal component analysis scores for cartilage (gray), bone (pink), and synovium (blue) samples measured on day 2 (circles) and day 7 (squares) of monocultures. 95% confidence intervals are shown as shades around the data points. **b** Dendrogram plot of sample hierarchical clustering analysis using complete linkage algorithm and Euclidean distance. **c** Heatmap visualization of relative abundance values of the measured metabolites. Statistically significant differences in metabolites between the monocultures are shown in bold (*p* < 0.05). Complete linkage hierarchical clustering with Euclidean distance was used for the dendrogram of metabolites

Metabolic signatures of the monocultures and tissue specificity of the detected metabolites were studied using the spent media samples collected on days 2 and 7 of cartilage, bone, and synovium monocultures from donors #1, 2, 3, and 7. Principal component analysis (Fig. 7a) and hierarchical clustering analysis (Fig. 7b) performed on the multi-donor dataset showed that cartilage and bone samples were more closely clustered, while synovium samples clustered separately and showed higher donor variability. Based on ANOVA results, 34 metabolites were found at differential relative abundance with statistical significance between the monocultures, and metabolite profiles in each monoculture were mostly conserved over time (Fig. 7c). A group of metabolites was enriched in synovium with lower detected levels in cartilage or bone samples, signifying synovium tissue specificity for these circulating metabolites. Out of the 9 metabolites that were significantly different between cartilage and bone samples, three TCA cycle compounds, citric acid, isocitric acid, and cis-aconitic acid, were depleted in cartilage samples.

### Synovium induces inflammatory response in osteochondral tissue cultures

**Fig. 8:**
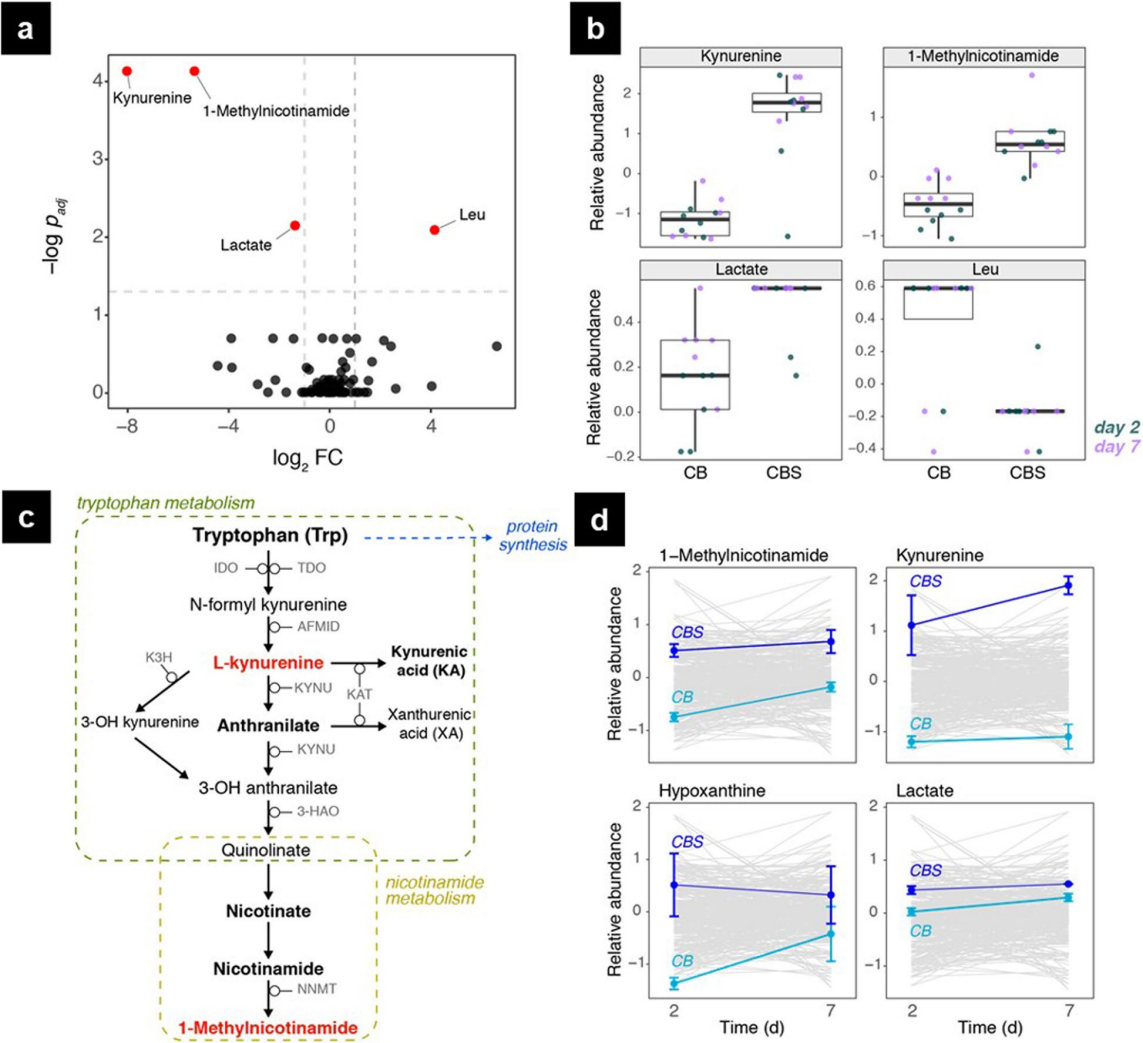
The effect of synovium on osteochondral plugs was assessed by comparing metabolic signatures in CB and CBS cocultures from donors #1, 7, 11, and 16. **a** Volcano plot of measured metabolites in spent media of CB and CBS cocultures with statistically significant metabolites marked in red and labeled. Fold change was calculated as metabolite abundance ratios in CB:CBS cocultures. **b** Relative abundance of the altered metabolites measured on day 2 (green) and day 7 (purple) in CB and CBS groups. **c** Simplified pathway maps of tryptophan and nicotinamide metabolism. Metabolites measured in this study are shown in bold and two metabolites altered in the CBS cocultures are shown in red. **d** Temporal abundance changes in metabolites significantly altered with respect to culture condition and time. Results are shown as mean ± SE. Changes in all measured metabolites are shown in gray in the background. CB, intact osteochondral plugs; CBS, osteochondral plugs cocultured with synovium; IDO, indoleamine 2,3-dioxygenase; TDO, tryptophan 2,3-dioxygenase; AFMID, arylformamidase; KYNU, kynureninase; KAT, kynurenine/2-aminoadipate aminotransferase; K3H, kynurenine 3-monooxygenase; 3-HAO, 3-hydroxyanthranilate 3,4-dioxygenase; NNMT, nicotinamide N-methyltransferase

To further study the effects of synovium on osteochondral plugs, circulating metabolites measured in CB and CBS cocultures of donors #1, 7, 11, and 16 were compared. Four metabolites that were significantly altered in CBS groups (*p* < 0.05) (Fig. 8a). Kynurenine, 1-methylnicotinamide, and lactate levels were elevated, while Leu levels were decreased in the CBS groups compared to the CB groups (Fig. 8b). The kynurenine pathway (Fig. 8c) is regulated by inflammatory cytokines and has been associated with inflammatory neurological diseases [30, 31] and RA and osteoporosis [32]. Connected to the kynurenine pathway, nicotinamide metabolism (Fig. 8c) has also been associated with rheumatoid arthritis (RA) and is possibly altered with chronic inflammation [33]. When the effects of culture condition and time were decoupled, the results showed that 1-methylnicotinamide, kynurenine, and hypoxanthine were significantly altered with respect to culture condition only, whereas the change in lactate levels was significant over time as well (Fig. 8d). Both lactate and hypoxanthine are elevated under hypoxia and indicate tissue injury [34].

To investigate the tissue specificity of altered metabolites, measured levels were compared in the spent media samples of cartilage, bone, and synovium monocultures from donors #1, 2, 3, and 7. In agreement with the measured elevation in CBS groups, kynurenine, 1-methylnicotinamide, and hypoxanthine levels were significantly higher in synovium samples compared to cartilage and bone monocultures (Supplementary Fig. S3). While not reaching statistical significance, mean lactate levels trended higher and Leu levels lower in synovium samples compared to cartilage and bone, consistent with the observed alterations in CB and CBS groups.

## Discussion

To our knowledge, this is the first in vitro model combining coculture of human knee osteochondral plugs with synovium explants obtained from the same donor-derived knees and incorporating an impact injury to the cartilage surface of the osteochondral plugs, all to aid in the study of pathways of initiation and earliest progression of PTOA. This model allows for crosstalk between cartilage, bone, and synovium over a 2-week culture period and the combined effects of mechanical injury with inflammation. We hypothesized that a model utilizing tissues from donors with no known history of joint disease could reveal the earliest changes in tissue properties and highlight early changes in biochemical and metabolite biomarkers. These events could then be compared with patient-derived synovial fluid markers [35] as well as clinical observations soon after a joint injury [36]. Such a model can also be used to study potential therapeutics and appropriate methods for drug delivery to specific tissues as soon as possible after injury [37].

We observed an immediate increase in the release of both ARGS-aggrecan fragments and associated sGAG into the medium over 2 weeks in CBS and CBS+INJ cocultures compared to CB-controls (Fig. 6, Tables S4 and S5). This was associated with the immediate release of inflammatory cytokines primarily from synovium (Figs. 3 and 4). Impact injury and inflammation, together, exacerbated loss of viable chondrocytes, an important early component of PTOA-like progression [38] (Fig. 2). The release of unique metabolites from cartilage, bone, and synovium monocultures, together with a group of circulating metabolites, also suggested synovium induction of an inflammatory response in osteochondral tissues (Figs. 7 and 8).

Since joint trauma often causes injurious mechanical impact to cartilage [5], we included clinically relevant compression of osteochondral plugs in our CBS+INJ treatment. In vitro models of injurious compression utilized by many groups involve specification of either peak stress or peak strain, along with the rate of loading [39]. Since cartilage thickness varied among our intact osteochondral plugs, it was not feasible to control peak strain for each individual plug within the culture environment; thus, we imposed peak stress. The applied loading rate is also critically important: when loaded slowly, cartilage tissue redistributes loads internally due to the fluid movement and realignment of matrix molecules (i.e., poroelastic relaxation) [40], thus preventing damage to the articular surface. However, at loading rates much higher than this fluid-matrix poroelastic redistribution time, fluid pressure and mechanical stress build up internally, which can cause widespread cell death and structural tissue damage. Taken together, we found that an applied loading rate of 15 MPa/s, up to a peak stress of 5 MPa, could successfully simulate increased cell death predominantly in the superficial zone (Fig. 2m, n) without gross damage, similar to the results of previous in vitro injury studies using human knee cartilage explants [39] and adult bovine osteochondral plugs [40], as well as observations of patient-derived osteochondral biopsies after ACL rupture [41]. Cartilage structural changes to an ellipsoidal shape or fissured appearance were observed in only a few samples, consistent with previous observations for these injurious loading conditions [39]. However, even in the absence of gross, visual damage to cartilage in vitro, or in vivo post-ACL injury [41], such injurious compression can induce microdamage within the cartilage matrix that could enhance transport of inflammatory cytokines from the surrounding media (or synovial fluid) into the cartilage, thereby accelerating matrix catabolism [42].

PTOA is a multi-tissue disease, and synovial macrophages, synoviocytes, and chondrocytes are all capable of expressing and secreting a range of inflammatory factors [43, 44]. In our study, we found the synovium to be the primary tissue source of inflammatory cytokines during the 2-week coculture (Figs. 3 and 4), consistent with the known central role of synovial inflammation and synovitis after traumatic human knee injuries [44]. We observed an immediate release of IL-1β, IL-6, TNF-α, IL-8, IFNγ, IL-12, and IL-13, along with IL-4, IL-10, and IL-2 in CBS and S explant cultures (e.g., Fig. 3). We note that cutting the synovium into 4–5-mm full-thickness explants may constitute a partial injury at the cut edges that could be responsible, in part, for the increased released of inflammatory cytokines. Concentrations of IL-1β, TNF-α, IL-6, and IFNγ in 2-week S, CBS, and CBS+INJ explant cultures from all donors taken together were significantly higher than those in C or CB cultures (Fig. 4). Importantly, synovial fluids from ACL-injured patients have also showed statistically significant increases in IL-6, TNF-α, IL-8, IFNγ, and IL-10 concentrations within the first days and weeks after injury, compared to reference (non-injured) subjects [7, 45, 46]. Interestingly, IL-6 concentrations in our CBS and CBS+INJ treatments are often ~100-fold higher than concentrations of any other cytokine. This marked increased in IL-6 release has also been found in ACL-injured synovial fluids, as high as 1000-fold compared to non-injured knees [7], further supporting the relevance of our CBS and CBS+INJ treatment models to human joint injuries. At the same time, it is clear that there is significant donor-to-donor variation in the levels of released cytokines (Fig. 4). In addition to mechanical injury to the articular cartilage and the damage to the bone, traumatic injuries to other associated tissues in the knee joint such as ACL rupture and meniscal tears are also strongly associated with the risk of developing OA [5]. However, these tissues were beyond the scope of this study, partly to limit the complexity and presence of confounding variables in the model developed here. The possibility of obtaining synovial fluid samples from such donor knees in future studies could also be explored to establish the baseline levels of cytokines and metabolites in the knee joints.

It is well known that traumatic knee injury in humans leads to increased levels of proteases and cartilage matrix fragments released to the synovial fluid associated with matrix degradation [47–49] including increased GAG loss. We saw an immediate increase in the loss of sGAG from the cartilage in response to coculture with synovium (a source of inflammatory mediators) and the combination of synovium and mechanically injury cartilage. The corresponding tissue GAG content was significantly lower in the CBS and CBS + INJ groups, as early as day 2. A rapid release of ARGS-aggrecan has previously been observed in synovial fluids of injured knees compared to reference subjects within days after ACL rupture [7]. We also observed significantly higher release of ARGS neoepitope fragments in the media of CBS and CBS+INJ groups compared to CB controls at days 2 and 7 of coculture (Fig. 6d), corresponding to aggrecanase activity in these treatment groups (and corresponding accumulated GAG loss (Fig. 6b)). At the same time, we did not detect increased release of CTX-II fragments (Fig. S2d) in CBS cultures or increased loss of total collagen (Fig. S2c) in CBS and CBS+INJ cultures. While CTX-II has been reported in urine samples after ACL rupture, it was suggested that this may be more closely related to turnover of calcified cartilage and bone, and not turnover of hyaline cartilage [7]. The delayed appearance of collagen breakdown markers in our in vitro studies may reflect the kinetics of the earliest features of cartilage proteolysis after joint injury and accompanying biomarker release to synovial fluids. Previous studies of isolated cartilage explants challenged with exogenous cytokines have shown similarly that collagen network degradation and loss does not begin until there is marked loss of aggrecan constituents and the presence of aggrecan may therefore protect cartilage collagen from proteolysis [50]. Thus, aggrecan proteolysis may be one of the earliest events (and therapeutic targets) in PTOA-associated cartilage breakdown.

Understanding metabolic changes in OA facilitates the discovery of biomarkers and supports the development of disease-modifying drugs. We focused on circulating metabolites for this investigation of early biomarker candidates in PTOA to understand disease initiation and progression. Various metabolomics studies using patient synovium tissue [51], synovial fluid [52], or serum [53] samples have provided extensive knowledge and diagnostic marker candidates, such as serum branched-chain amino acid-to-histidine ratio. However, there is no complete agreement on altered metabolites or the molecular signature of OA due to heterogeneity of the disease and patient variability. Despite this, the majority of these studies pointed to a biochemical signature associated with hypoxia, inflammation, and high energy requirements suggested by observed perturbations in metabolic pathways [54]. Consistent with these studies, our results showed metabolite alterations related to hypoxia and inflammation, in addition to perturbations in amino acid metabolism. Common limitations in previous OA metabolomics studies, such as using animal models or lack of balanced patient population, were mitigated here by using human tissue cultures from multiple donors with no previously known OA history. Nevertheless, our results demonstrated donor variability and suggested donor specificity for some metabolites, such as kynurenine, which was elevated in CBS and CBS+INJ, but was not detected in some donors.

Limitation of this study centered around donor availability and the total number of CB and S samples that could be harvested from any single donor joint. The earliest stages of PTOA are believed to result from the combination of initial mechanical trauma accompanied by release of inflammatory cytokines to the bathing synovial fluid. Thus, after our initial series of experiments to characterize the monocultures, our experimental design for cocultures focused on the CBS+INJ condition as the closest representation of the PTOA disease environment, requiring 5–6 technical replicates (tissues) for each treatment condition, at each time point studied, per available knee. Due to total sample limitations, we could not pursue as many CBS-alone treatments as might be desirable or test an injury-only condition for comparison (i.e., CB+INJ), especially since some assays (e.g., histopathology, biochemistry) are destructive, which required separate sets of tissue groups for each time point studied. Donor variability was exemplified, for example, by the wide range of measured values for concentrations of released cytokines (Fig. 4) and aggrecan ARGS neoepitope fragments (Fig. 6d). Donor variability may also be the underlying reason for the spread of values of GAG and total protein biosynthesis (Fig. S2a, b), for which no trends were observed. However, such variability is inherent to clinical studies of patients, including those who have sustained ACL injuries. Indeed, Jacobs et al. [35] found separate clusters of high (“dysregulated”) and low inflammatory responses associated with synovial fluid levels of cytokines, degradative enzymes, and biomarkers of cartilage degradation; this variability was not a function of injury severity. Thus, donor-to-donor differences in our in vitro tissues likely reflect similar variability among tissues from knees of different patients. Further studies are needed to see if age, gender, and initial starting tissue grade are determinants of these differences.

Despite this variability, we found that, using a coculture model which simulates the relationship between osteochondral tissue and synovium in the human joint, we observed critical early inflammatory response markers produced by synovial tissue. These markers included multiple cytokines and metabolites that may play a role in the etiopathogenesis of PTOA. The data further demonstrate that these synovial products promote the degradation of articular cartilage. This degradation is superimposed on the loss of cartilage cellularity and cellular viability imposed by injurious compression. Interestingly, the available data comparing *CBS* and *CBS+INJ* cultures provide little evidence for a synergistic interaction between synovial inflammatory markers and injurious compression in promoting cartilage deterioration. This may reflect the very early phase of the disease process represented in these experiments. However, recent proteomic studies of the kinetics of disease biomarker release [55] also suggest that a single mechanical impact injury can cause matrix breakdown to begin several days sooner than that caused by inflammation alone. This may be associated with injury-induced microdamage to the matrix enabling enhanced transport of mediators into cartilage and the ensuing release of matrix breakdown products.

## Conclusion

The new osteochondral-synovium coculture model developed and studied here offers several advantages over other models of PTOA. The use of human knee tissues from donors that had no prior history of OA disease suggests the relevance of this model to the earliest stages of PTOA progression. The CBS+INJ system, in particular, shows distinct cellular, inflammatory, and matrix-related alterations relevant to PTOA-like initiation and progression. The use of human synovium provides an endogenous source of cytokines and metabolites rather than providing a predetermined cocktail of exogenous inflammatory molecules that is unlikely to reproduce synovial production. Additionally, models which employ human osteochondral samples that maintain the attachment of articular cartilage to subchondral bone provide a superior simulation of magnitude and distribution of mechanical stress in vitro and a better correlation between loading parameters and the cellular response to trauma. Ongoing studies using this coculture model involve mass spectroscopy proteomic analyses, studies of the effects of dexamethasone and insulin-like growth factor-1 on disease modification, and the effects of radiation and microgravity in low earth orbit found on the International Space Station.

## Supplementary Information

**Additional file 1: Supplementary Table S1.** Summary of human donor joints used in the study. 25 cadaveric knees from 16 donors were classified as Collin’s grade 0 (normal, *n* = 1), grade 1 (near normal, *n* = 8) and grade 2 (fibrillation in some delimited regions, *n* = 16). Donors age 23-83 yo; 8 females, 8 males. Groups studied: C – cartilage monocultures; B – Bone monocultures; S – monocultures of full thickness explant tissue inclusive of synovium and fibrous joint capsule, referred to as synovium S for simplicity; CB – intact osteochondral plugs; CBS – osteochondral plugs cocultured with synovium S; CBS + INJ – Mechanically injured osteochondral plugs (CB) cocultured with synovium to simulate PTOA-disease like condition. The “Groups” column lists treatment groups that were chosen to be tested for each donor, based on the total available tissues that could be harvested from each knee.

**Additional file 2: Supplementary Fig. S1.** Experimental design and sample collection time points. Following a 2-day pre-equilibration after the tissue harvest, experiments started on day 0. Experiments included cartilage, bone and synovium monocultures (C, B, S), osteochondral (cartilage-bone) plug cultures (CB), osteochondral plugs cocultured with synovium (CBS). The cultures were maintained for a period of 4 weeks for initial experiments comparing C, B, S monocultures, CB and CBS coculture conditions (donors # 1-7). Since introduction of injury adversely impacted the viability at much earlier time point, the cultures were terminated at 2 weeks in the later experiments comparing CB, CBS and CBS+INJ conditions (donors # 8-16). For the condition CBS+INJ, the cartilage surface of osteochondral plugs was first subjected to a single compressive impact injury and then CB was cocultured with synovium explants S. Spent media (SM) was collected at every media change and tissue samples (T) were collected weekly for determination of viability, metabolic, and biochemical alterations.

**Additional file 3: Supplementary Table S2.** Analysis of Fig. 4 Data. Group differences in the total amount (over 14-days) of secreted cytokines analyzed using donor-matched mixed-effects model (REML). False discovery rate (FDR, Benjamini Hochberg method) was used to adjust for multiple comparisons. Only statistically significant (*q < 0.05*) comparisons are tabulated for each cytokine measured.

**Additional file 4: Supplementary Table S3.** Further Analysis of Fig. 4 Data. Analysis of group differences in secreted cytokines TNF-α, IL-1, IFNγ, and IL-6, over 14 days in mono- or cocultures. Time point-matched mixed-effects (REML) analysis was performed with Geisser-Greenhouse correction for sphericity. False discovery rate (FDR, Benjamini Hochberg method) was used to adjust for multiple comparisons. Only statistically significant (*q < 0.05*) comparisons are tabulated for each cytokine measured.

**Additional file 5: Supplementary Table S4.** Analysis of pairwise comparisons of differences in GAG content from Fig. 6b. Analysis of pairwise comparisons of differences in tissue GAG content between groups shown in Fig. 6b. The significant differences have been noted with + for C vs CBS and + for C vs CBS+INJ. The significant difference between CB vs CBS have been marked with # and # for CB vs CBS+INJ.

**Additional file 6: **S**upplementary Table S5.** Analysis of Fig 6d data. Summary of Wilcoxon rank sum test results for ARGS-aggrecan release for conditions with significant differences. Data are grouped by condition and day.

**Additional file 7: Supplementary Fig. S2.** (a) Rate of biosynthesis of sGAG and (b) total protein was measured in monoculture and cocultures at week (W) 1, 2, 3 and 4. (c) Changes in collagen content were measured in cartilage tissue in response to coculture with inflammatory cytokines and mechanical impact injury. (d) Release of collagenase generated CTX-II fragments was measured in pooled spent media samples on day 2, 7 and 14 of culture to assess early changes in collagen degradation. C – Cartilage monocultures (*N* = 5); CB – intact osteochondral plugs (*N* = 10); CBS - osteochondral plugs cocultured with synovium (*N* = 5); CBS + INJ – Mechanically injured osteochondral plugs cocultured with synovium (*N* = 6).

**Additional file 8: Supplementary Fig. S3.** Significantly altered metabolites in CB and CBS group comparison examined in monocultures. Circulating metabolites measured in cartilage, bone, and synovium monocultures from donors # 1, 2, 3, and 7 on days 2 (green) and 7 (purple).

**Additional file 9.** Supplemental Methods.

## Data Availability

All data are included in this manuscript and Supplementary Materials. These data will also be posted on our laboratory website.

## Abbreviations

S: Synovium
C: Cartilage
CB: Cartilage-bone cocultures
CBS: Cartilage-bone-synovium cocultures
CBS + INJ: Cartilage-bone-synovium cocultures with mechanically injured cartilage
ACL: Anterior cruciate ligament
PTOA: Post-traumatic osteoarthritis
MPa: Megapascal
OA: Osteoarthritis
RA: Rheumatoid arthritis
sGAG: Sulfated glycosaminoglycans
DMMB: Dimethylmethylene blue
IL: Interleukin
TNF α: Tumor necrosis factor α
IFN γ: Interferon γ
FDA: Fluorescein diacetate
PI: Propidium iodide
MTT: 3-(4,5-Dimethylthiazol-2-yl)-2,5-diphenyltetrazolium bromide
H&E: Hematoxylin and eosin
OHP: Hydroxyproline
CTX II: C-telopeptide fragments of type II collagen
CE-TOFMS: Capillary electrophoresis time-of-flight mass spectrometry
IDO: Indoleamine 2,3-dioxygenase
TDO: Tryptophan 2,3-dioxygenase
AFMID: Arylformamidase
KYNU: Kynureninase
KAT: Kynurenine/2-aminoadipate aminotransferase
K3H: Kynurenine 3-monooxygenase
3-HAO: 3-Hydroxyanthranilate 3,4-dioxygenase
NNMT: Nicotinamide N-methyltransferase
