# Supplementary material for: Inflammatory cytokines and mechanical injury induce post-traumatic osteoarthritis-like changes in a human cartilage-bone-synovium microphysiological system
Source: Arthritis Res Ther. 2022 Aug 18;24:198. doi: 10.1186/s13075-022-02881-z (PMC9386988; doi:10.1186/s13075-022-02881-z)
Supplement: Supplementary file 1 — Additional file 1: Supplementary Table S1. Summary of human donor joints used in the study. 25 cadaveric knees from 16 donors were classified as Collin’s grade 0 (normal, n = 1), grade 1 (near normal, n = 8) and grade 2 (fibrillation in some delimited regions, n = 16). Donors age 23-83 yo; 8 females, 8 males. Groups studied: C – cartilage monocultures; B – Bone monocultures; S – monocultures of full thickness explant tissue inclusive of synovium and fibrous joint capsule, referred to as synovium S for simplicity; CB – intact osteochondral plugs; CBS – osteochondral plugs cocultured with synovium S; CBS + INJ – Mechanically injured osteochondral plugs (CB) cocultured with synovium to simulate PTOA-disease like condition. The “Groups” column lists treatment groups that were chosen to be tested for each donor, based on the total available tissues that could be harvested from each knee. [file 13075_2022_2881_MOESM1_ESM.docx]

**Supplementary Table S1:** **Summary of human donor joints used in the study.** 25 cadaveric knees from 16 donors were classified as Collin’s grade 0 (normal, n=1), grade 1 (near normal, n=8) and grade 2 (fibrillation in some delimited regions, n=16). Donors age 23-83 yo; 8 females, 8 males. Groups studied: **C** – cartilage monocultures; **B** – Bone monocultures; **S** – monocultures of full thickness explant tissue inclusive of synovium and fibrous joint capsule, referred to as synovium **S** for simplicity; **CB** – intact osteochondral plugs; **CBS** – osteochondral plugs cocultured with synovium **S**; **CBS + INJ** – Mechanically injured osteochondral plugs (CB) cocultured with synovium to simulate PTOA-disease like condition. The “Groups” column lists treatment groups that were chosen to be tested for each donor, based on the total available tissues that could be harvested from each knee.

| **Donor No.** | **Knee No.** | **Sex** | **Age** | **Grade** | **Groups** | **Outcome measurement** |
| --- | --- | --- | --- | --- | --- | --- |
| 1 | 1 | M | 81 | 2 | C B S CB CBS | Biochemistry, Inflammation, Metabolomics, Histology |
|  | 2 |  |  | 2 |  |  |
| 2 | 3 | F | 41 | 1 | C B S CB | Biochemistry, Inflammation, Metabolomics, Histology |
|  | 4 |  |  | 1 |  |  |
| 3 | 5 | M | 76 | 2 | C B S CBS | Biochemistry, Inflammation, Metabolomics, Histology |
|  | 6 |  |  | 2 |  |  |
| 4 | 7 | M | 83 | 1 | C B S CB | Biochemistry, Inflammation |
| 5 | 8 | F | 72 | 2 | CB | Biochemistry, Inflammation |
|  | 9 |  |  | 2 |  |  |
| 6 | 10 | F | 76 | 2 | CBS | Biochemistry, Inflammation, Histology |
|  | 11 |  |  | 1 |  |  |
| 7 | 12 | F | 76 | 2 | C B S CB CBS | Biochemistry, Inflammation, Metabolomics, Histology |
|  | 13 |  |  | 2 |  |  |
| 8 | 14 | M | 67 | 2 | CBS CBS + INJ | Biochemistry, Inflammation |
| 9 | 15 | F | 75 | 2 | CBS + INJ | Biochemistry, Inflammation |
|  | 16 |  |  | 2 |  |  |
| 10 | 17 | F | 79 | 2 | CBS + INJ | Biochemistry, Inflammation |
| 11 | 18 | F | 78 | 2 | CB CBS CBS + INJ | Biochemistry, Inflammation, Metabolomics, Histology |
|  | 19 |  |  | 2 |  |  |
| 12 | 20 | M | 79 | 1 | CB CBS + INJ | Biochemistry, Inflammation, Metabolomics, Histology |
|  | 21 |  |  | 1 |  |  |
| 13 | 22 | M | 34 | 0 | CB CBS + INJ | Biochemistry, Inflammation, Metabolomics, Histology |
| 14 | 23 | M | 23 | 1 | CB CBS + INJ | Biochemistry, Inflammation, Metabolomics, Histology |
| 15 | 24 | M | 54 | 2 | CB CBS + INJ | Biochemistry, Inflammation, Histology |
| 16 | 25 | F | 58 | 1 | CB CBS + INJ | Biochemistry, Inflammation, Metabolomics, Histology |
