# Supplementary figures and images for: Inflammatory cytokines and mechanical injury induce post-traumatic osteoarthritis-like changes in a human cartilage-bone-synovium microphysiological system
Source: Arthritis Res Ther. 2022 Aug 18;24:198. doi: 10.1186/s13075-022-02881-z (PMC9386988; doi:10.1186/s13075-022-02881-z)

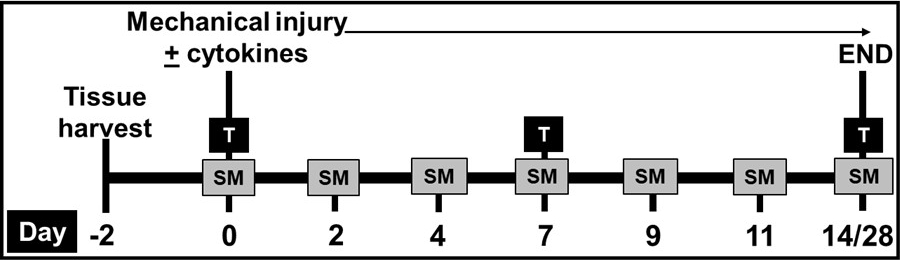

Supplement: Supplementary file 2 — Additional file 2: Supplementary Fig. S1. Experimental design and sample collection time points. Following a 2-day pre-equilibration after the tissue harvest, experiments started on day 0. Experiments included cartilage, bone and synovium monocultures (C, B, S), osteochondral (cartilage-bone) plug cultures (CB), osteochondral plugs cocultured with synovium (CBS). The cultures were maintained for a period of 4 weeks for initial experiments comparing C, B, S monocultures, CB and CBS coculture conditions (donors # 1-7). Since introduction of injury adversely impacted the viability at much earlier time point, the cultures were terminated at 2 weeks in the later experiments comparing CB, CBS and CBS+INJ conditions (donors # 8-16). For the condition CBS+INJ, the cartilage surface of osteochondral plugs was first subjected to a single compressive impact injury and then CB was cocultured with synovium explants S. Spent media (SM) was collected at every media change and tissue samples (T) were collected weekly for determination of viability, metabolic, and biochemical alterations. [file 13075_2022_2881_MOESM2_ESM.jpg]

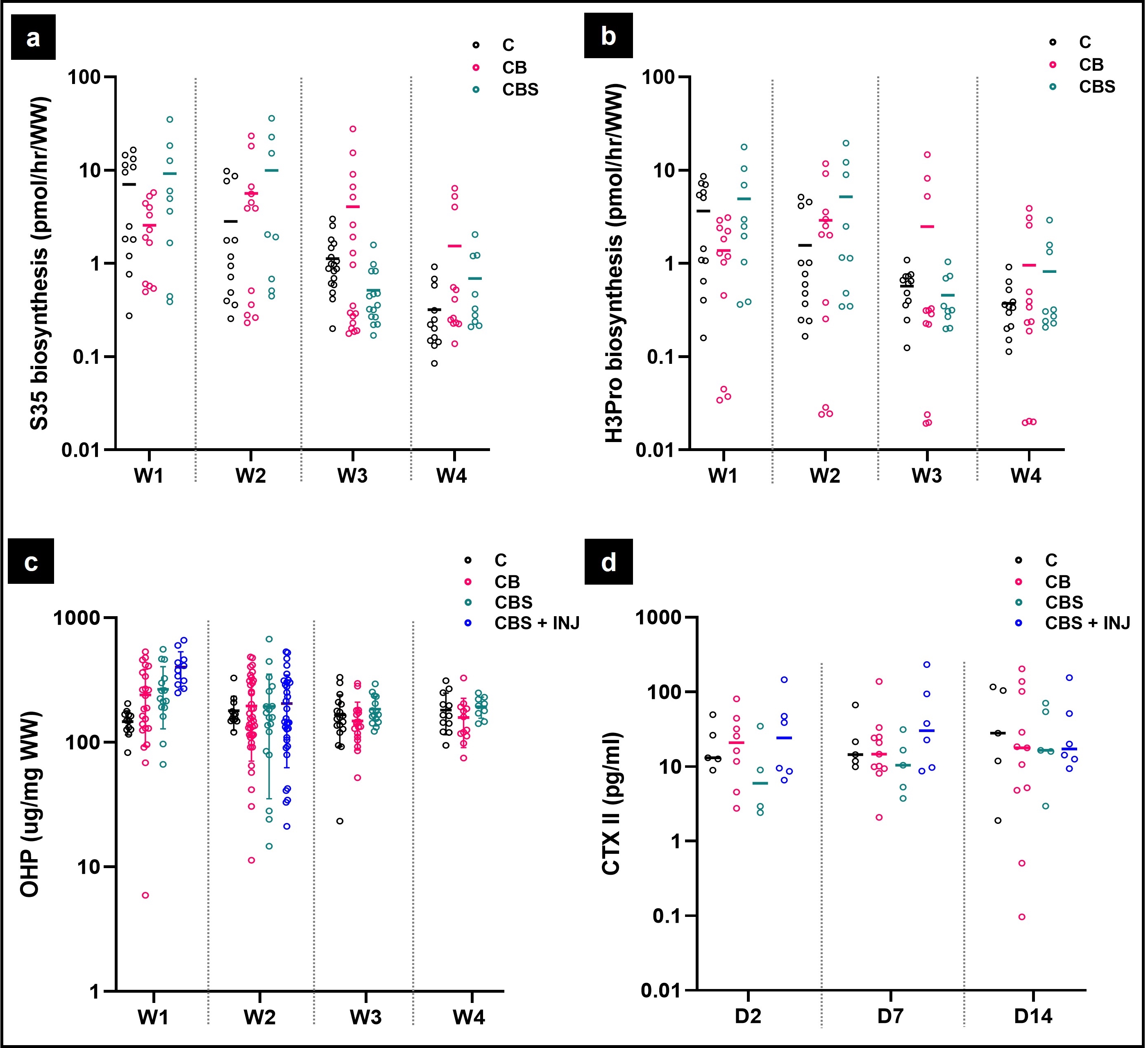

Supplement: Supplementary file 7 — Additional file 7: Supplementary Fig. S2. (a) Rate of biosynthesis of sGAG and (b) total protein was measured in monoculture and cocultures at week (W) 1, 2, 3 and 4. (c) Changes in collagen content were measured in cartilage tissue in response to coculture with inflammatory cytokines and mechanical impact injury. (d) Release of collagenase generated CTX-II fragments was measured in pooled spent media samples on day 2, 7 and 14 of culture to assess early changes in collagen degradation. C – Cartilage monocultures (N = 5); CB – intact osteochondral plugs (N = 10); CBS - osteochondral plugs cocultured with synovium (N = 5); CBS + INJ – Mechanically injured osteochondral plugs cocultured with synovium (N = 6). [file 13075_2022_2881_MOESM7_ESM.jpg]

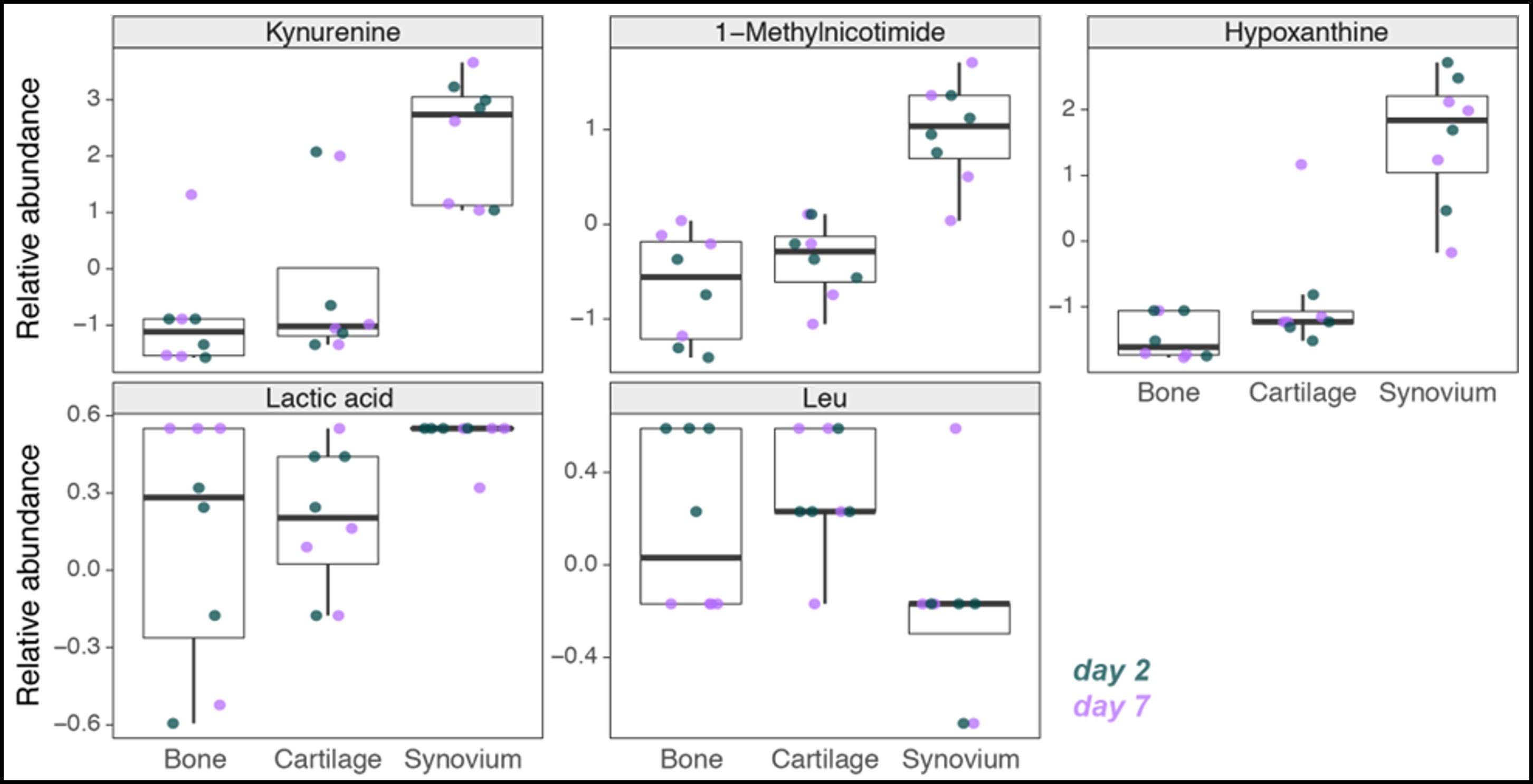

Supplement: Supplementary file 8 — Additional file 8: Supplementary Fig. S3. Significantly altered metabolites in CB and CBS group comparison examined in monocultures. Circulating metabolites measured in cartilage, bone, and synovium monocultures from donors # 1, 2, 3, and 7 on days 2 (green) and 7 (purple). [file 13075_2022_2881_MOESM8_ESM.jpg]
