# Supplementary material for: Inflammatory cytokines and mechanical injury induce post-traumatic osteoarthritis-like changes in a human cartilage-bone-synovium microphysiological system
Source: Arthritis Res Ther. 2022 Aug 18;24:198. doi: 10.1186/s13075-022-02881-z (PMC9386988; doi:10.1186/s13075-022-02881-z)
Supplement: Supplementary file 3 — Additional file 3: Supplementary Table S2. Analysis of Fig. 4 Data. Group differences in the total amount (over 14-days) of secreted cytokines analyzed using donor-matched mixed-effects model (REML). False discovery rate (FDR, Benjamini Hochberg method) was used to adjust for multiple comparisons. Only statistically significant (q < 0.05) comparisons are tabulated for each cytokine measured. [file 13075_2022_2881_MOESM3_ESM.docx]

**Supplementary Table S2: Analysis of Fig. 4 Data.** Group differences in the total amount (over 14-days) of secreted cytokines analyzed using donor-matched mixed-effects model (REML). False discovery rate (FDR, Benjamini Hochberg method) was used to adjust for multiple comparisons. Only statistically significant (*q < 0.05*) comparisons are tabulated for each cytokine measured.

| **TNF-α total release** | | | | |
| --- | --- | --- | --- | --- |
| **Fixed effect (type III)** | ***p-value*** | **(*p < 0.05*)?** | **F (DFn, DFd)** |  |
| Treatment (culture) | 0.0071 | Yes | F (4, 17) = 5.069 |  |
| **Multiple comparisons** | **Mean Diff.** | **SE of diff.** | ***q-value*** | ***p-value*** |
| CBS vs. C | 186.8 | 54.86 | 0.0169 | 0.0034 |
| CB vs. CBS | -173.9 | 46.48 | 0.0163 | 0.0016 |
| CBS+INJ vs. CB | 123.3 | 42.91 | 0.0352 | 0.0105 |
| **IL-1 total release** | | | | |
| **Fixed effect (type III)** | ***p-value*** | **(*p < 0.05*)?** | **F (DFn, DFd)** |  |
| Treatment (culture) | 0.0730 | No | F (4, 17) = 2.601 |  |
| **IFNγ total release** | | | | |
| **Fixed effect (type III)** | ***p-value*** | **(*p < 0.05*)?** | **F (DFn, DFd)** |  |
| Treatment (culture) | 0.0041 | Yes | F (4, 17) = 5.748 |  |
| **Multiple comparisons** | **Mean Diff.** | **SE of diff.** | ***q-value*** | ***p-value*** |
| CBS vs. C | 326.6 | 82.65 | 0.0052 | 0.0010 |
| CB vs. S | 268.8 | 82.65 | 0.0156 | 0.0047 |
| CB vs. CBS | -299.1 | 70.36 | 0.0052 | 0.0005 |
| **IL-6 total release** | | | | |
| **Fixed effect (type III)** | ***p-value*** | **(*p < 0.05*)?** | **F (DFn, DFd)** |  |
| Treatment (culture) | 0.3110 | No | F (4, 17) = 1.296 |  |
