# Supplementary material for: Inflammatory cytokines and mechanical injury induce post-traumatic osteoarthritis-like changes in a human cartilage-bone-synovium microphysiological system
Source: Arthritis Res Ther. 2022 Aug 18;24:198. doi: 10.1186/s13075-022-02881-z (PMC9386988; doi:10.1186/s13075-022-02881-z)
Supplement: Supplementary file 4 — Additional file 4: Supplementary Table S3. Further Analysis of Fig. 4 Data. Analysis of group differences in secreted cytokines TNF-α, IL-1, IFNγ, and IL-6, over 14 days in mono- or cocultures. Time point-matched mixed-effects (REML) analysis was performed with Geisser-Greenhouse correction for sphericity. False discovery rate (FDR, Benjamini Hochberg method) was used to adjust for multiple comparisons. Only statistically significant (q < 0.05) comparisons are tabulated for each cytokine measured. [file 13075_2022_2881_MOESM4_ESM.docx]

**Supplementary Table S3: Further Analysis of Fig. 4 Data**. Analysis of group differences in secreted cytokines TNF-α, IL-1, IFNγ, and IL-6, over 14 days in mono- or cocultures. Time point-matched mixed-effects (REML) analysis was performed with Geisser-Greenhouse correction for sphericity. False discovery rate (FDR, Benjamini Hochberg method) was used to adjust for multiple comparisons. Only statistically significant (*q < 0.05*) comparisons are tabulated for each cytokine measured.

| **TNF-α release** | | | | |
| --- | --- | --- | --- | --- |
| **Fixed effect (type III)** | ***p-value*** | **(*p < 0.05*)?** | **F (DFn, DFd)** | **Geisser-Greenhouse's epsilon** |
| Treatment (culture) | 0.0218 | Yes | F (1.719, 10.31) = 5.978 | 0.4297 |
| **Multiple comparisons** | **Mean Diff.** | **SE of diff.** | ***q-value*** | ***p-value*** |
| CBS vs. C | 21.98 | 3.131 | 0.0045 | 0.0009 |
| CB vs. C | -3.724 | 0.9563 | 0.0229 | 0.0115 |
| CBS+INJ vs. C | 23.94 | 6.107 | 0.0229 | 0.0112 |
| CB vs. CBS | -25.71 | 3.052 | 0.0007 | <0.0001 |
| CBS+INJ vs. CB | 27.66 | 6.434 | 0.0119 | 0.0036 |
| **IL-1 release** | | | | |
| **Fixed effect (type III)** | ***p-value*** | **(*p < 0.05*)?** | **F (DFn, DFd)** | **Geisser-Greenhouse's epsilon** |
| Treatment (culture) | 0.0021 | Yes | F (1.651, 9.906) = 13.29 | 0.4127 |
| **Multiple comparisons** | **Mean Diff.** | **SE of diff.** | ***q-value*** | ***p-value*** |
| S vs. C | 28.84 | 8.372 | 0.0305 | 0.0183 |
| CBS vs. C | 23.49 | 4.617 | 0.0106 | 0.0038 |
| CBS+INJ vs. C | 46.87 | 10.28 | 0.0121 | 0.0061 |
| CB vs. S | -34.19 | 6.893 | 0.0106 | 0.0042 |
| CB vs. CBS | -28.84 | 5.474 | 0.0106 | 0.0012 |
| CBS+INJ vs. CBS | 23.38 | 8.435 | 0.0395 | 0.0276 |
| CBS+INJ vs. CB | 52.21 | 12.50 | 0.0106 | 0.0041 |
| **IFNγ release** | | | | |
| **Fixed effect (type III)** | ***p-value*** | **(*p < 0.05*)?** | **F (DFn, DFd)** | **Geisser-Greenhouse's epsilon** |
| Treatment (culture) | 0.0045 | Yes | F (1.332, 7.993) = 13.52 | 0.3330 |
| **Multiple comparisons** | **Mean Diff.** | **SE of diff.** | ***q-value*** | ***p-value*** |
| CBS vs. C | 44.05 | 8.358 | 0.0146 | 0.0033 |
| CBS+INJ vs. C | 53.72 | 17.04 | 0.0422 | 0.0253 |
| CBS vs. S | 25.22 | 5.120 | 0.0146 | 0.0044 |
| CB vs. S | -23.44 | 6.671 | 0.0341 | 0.0170 |
| CB vs. CBS | -48.66 | 7.505 | 0.0034 | 0.0003 |
| CBS+INJ vs. CB | 58.33 | 16.11 | 0.0212 | 0.0085 |
| **IL-6 release** | | | | |
| **Fixed effect (type III)** | ***p-value*** | **(*p < 0.05*)?** | **F (DFn, DFd)** | **Geisser-Greenhouse's epsilon** |
| Treatment (culture) | 0.2192 | No | F (1.043, 6.259) = 1.875 | 0.2608 |
| **Multiple comparisons** | **Mean Diff.** | **SE of diff.** | ***q-value*** | ***p-value*** |
| S vs. C | 1.966 | 0.1759 | 0.0010 | 0.0001 |
| CB vs. S | -1.997 | 0.2485 | 0.0024 | 0.0005 |
