# Supplementary material for: Inflammatory cytokines and mechanical injury induce post-traumatic osteoarthritis-like changes in a human cartilage-bone-synovium microphysiological system
Source: Arthritis Res Ther. 2022 Aug 18;24:198. doi: 10.1186/s13075-022-02881-z (PMC9386988; doi:10.1186/s13075-022-02881-z)
Supplement: Supplementary file 5 — Additional file 5: Supplementary Table S4. Analysis of pairwise comparisons of differences in GAG content from Fig. 6b. Analysis of pairwise comparisons of differences in tissue GAG content between groups shown in Fig. 6b. The significant differences have been noted with + for C vs CBS and + for C vs CBS+INJ. The significant difference between CB vs CBS have been marked with # and # for CB vs CBS+INJ. [file 13075_2022_2881_MOESM5_ESM.docx]

**Supplementary Table S4: Analysis of pairwise comparisons of differences in GAG content from Fig. 6b.** Analysis of pairwise comparisons of differences in tissue GAG content between groups shown in Fig. 6b. The significant differences have been noted with + for C vs CBS and + for C vs CBS+INJ. The significant difference between CB vs CBS have been marked with # and # for CB vs CBS+INJ.

| **Group** | **Summary** | **P-values** |
| --- | --- | --- |
| **Day 2** | | |
| C vs. CB | ** | 0.0010 |
| C vs. CBS (+) | **** | <0.0001 |
| C vs. CBS+INJ (+) | **** | <0.0001 |
| CB vs. CBS (#) | ** | 0.0062 |
| CB vs. CBS+INJ (#) | **** | <0.0001 |
| CBS vs. CBS+INJ | ns | 0.5688 |
|  |  |  |
| **Day 4** | | |
| C vs. CB | ns | 0.2899 |
| C vs. CBS (+) | **** | <0.0001 |
| C vs. CBS+INJ (+) | **** | <0.0001 |
| CB vs. CBS (#) | **** | <0.0001 |
| CB vs. CBS+INJ (#) | **** | <0.0001 |
| CBS vs. CBS+INJ | ns | 0.3327 |
| **Day 7** | | |
| C vs. CB | ns | 0.4364 |
| C vs. CBS (+) | **** | <0.0001 |
| C vs. CBS+INJ (+) | **** | <0.0001 |
| CB vs. CBS (#) | **** | <0.0001 |
| CB vs. CBS+INJ (#) | **** | <0.0001 |
| CBS vs. CBS+INJ | ns | >0.9999 |
| **Day 9** | | |
| C vs. CB | ns | 0.1639 |
| C vs. CBS (+) | **** | <0.0001 |
| C vs. CBS+INJ (+) | **** | <0.0001 |
| CB vs. CBS (#) | **** | <0.0001 |
| CB vs. CBS+INJ (#) | **** | <0.0001 |
| CBS vs. CBS+INJ | ns | >0.9999 |
| **Day 11** | | |
| C vs. CB | ns | 0.1330 |
| C vs. CBS (+) | **** | <0.0001 |
| C vs. CBS+INJ (+) | **** | <0.0001 |
| CB vs. CBS (#) | **** | <0.0001 |
| CB vs. CBS+INJ (#) | **** | <0.0001 |
| CBS vs. CBS+INJ | ns | 0.9735 |
| **Day 14** | | |
| C vs. CB | ns | 0.2042 |
| C vs. CBS (+) | **** | <0.0001 |
| C vs. CBS+INJ (+) | **** | <0.0001 |
| CB vs. CBS (#) | **** | <0.0001 |
| CB vs. CBS+INJ (#) | **** | <0.0001 |
| CBS vs. CBS+INJ | ns | 0.9792 |
