# Supplementary material for: Inflammatory cytokines and mechanical injury induce post-traumatic osteoarthritis-like changes in a human cartilage-bone-synovium microphysiological system
Source: Arthritis Res Ther. 2022 Aug 18;24:198. doi: 10.1186/s13075-022-02881-z (PMC9386988; doi:10.1186/s13075-022-02881-z)
Supplement: Supplementary file 6 — Additional file 6: Supplementary Table S5. Analysis of Fig 6d data. Summary of Wilcoxon rank sum test results for ARGS-aggrecan release for conditions with significant differences. Data are grouped by condition and day. [file 13075_2022_2881_MOESM6_ESM.docx]

**Supplementary Table S5: Analysis of Fig 6d data.** Summary of Wilcoxon rank sum test results for ARGS-aggrecan release for conditions with significant differences. Data are grouped by condition and day.

| **Test Groups** | **Conditions with significant differences** | **P values** |
| --- | --- | --- |
| Overall results – for data grouped by condition | | |
|  | C and CBS + INJ | .0133 |
|  | CB and CBS | .0011 |
|  | CB and CBS + INJ | .0002 |
| Pair wise comparison for data grouped by condition | | |
| Day 2 | CB and CBS + INJ | .0312 |
| Day 7 | CB and CBS | .00266 |
|  | CB and CBS + INJ | .00524 |
| Day 14 | None | -- |
| Overall results – for data grouped by time point (day) | | |
|  | Day 2 and 14 | 0.0119 |
| Pair wise comparison for data grouped by timepoint (day) | | |
| C | None | -- |
| CB | None | -- |
| CBS | None | -- |
| CBS + INJ | None | -- |
