# Supplementary material for: Inflammatory cytokines and mechanical injury induce post-traumatic osteoarthritis-like changes in a human cartilage-bone-synovium microphysiological system
Source: Arthritis Res Ther. 2022 Aug 18;24:198. doi: 10.1186/s13075-022-02881-z (PMC9386988; doi:10.1186/s13075-022-02881-z)
Supplement: Supplementary file 9 — Additional file 9. Supplemental Methods. [file 13075_2022_2881_MOESM9_ESM.docx]

**Supplemental Methods**

***A. Harvesting Osteochondral plugs***

Mosaicplasty tool set (Smith & Nephew, Cat No. 7207098, Fig 1a) comprising of a mallet, 3.5mm diameter tubular chisel and harvesting tamps (2.7mm and 3.5mm) were used for harvesting of osteochondral plugs [Fig.1f]. The sharp edge of the tubular chisel was placed perpendicular to the cartilage surface of the knee joint. The mallet was then used to impact the chisel to a depth of 10-12 mm [Fig. 1d]. Harvesting tamp was then inserted into the chisel at the opposite end, parallel to the cartilage surface. Holding the harvesting tamp, the chisel was withdrawn from the joint by applying pressure in the opposite direction accompanied by wiggling motion to break away the osteochondral plug from the subchondral bone. The chisel was then placed on a sterile gauze, bone surface facing upward. Harvesting tamp (3.5mm) was placed perpendicular and flush against the surface of the bone and the pressure was applied using a mallet to release the osteochondral plug from the chisel.

***B. Measurement of ARGS-aggrecan fragments***

Five selected medium samples were chosen for performance a test-of-assay. Samples diluted between 2 and 10 times and spiked with standards showed good spiking-recovery (mean 96-115%, range 90-120%). Test medium samples (n = 5) diluted between 1.3 and 10.1 times showed no dilution-linearity (recovery at: 3x dilution = 170-183%, 6.8x dilution = 246-295%, 10.1x dilution = 285-365%). All medium samples were run in duplicates at 2.6X dilution.
